# Understanding salt tolerance mechanism using transcriptome profiling and de novo assembly of wild tomato *Solanum chilense*

**DOI:** 10.1038/s41598-020-72474-w

**Published:** 2020-09-28

**Authors:** S. P. Kashyap, H. C. Prasanna, Nishi Kumari, Pallavi Mishra, B. Singh

**Affiliations:** 1 Division of Crop Improvement and Biotechnology, Indian Institute of Vegetable Research, Indian Council of Agricultural Research, Varanasi, Uttar Pradesh 221 305 India; 2 Department of Botany, Mahila Maha Vidyalaya, Banaras Hindu University, Varanasi, Uttar Pradesh 221 005 India; 3 Division of Vegetable Crops, Indian Institute of Horticultural Research, Indian Council of Agricultural Research, Hessaraghatta, Lake Post, Bengaluru, Karnataka 560 089 India

**Keywords:** Biotechnology, Plant sciences

## Abstract

Soil salinity affects the plant growth and productivity detrimentally, but *Solanum chilense*, a wild relative of cultivated tomato (*Solanum lycopersicum* L.), is known to have exceptional salt tolerance. It has precise adaptations against direct exposure to salt stress conditions. Hence, a better understanding of the mechanism to salinity stress tolerance by *S. chilense* can be accomplished by comprehensive gene expression studies. In this study 1-month-old seedlings of *S. chilense* and *S. lycopersicum* were subjected to salinity stress through application of sodium chloride (NaCl) solution. Through RNA-sequencing here we have studied the differences in the gene expression patterns. A total of 386 million clean reads were obtained through RNAseq analysis using the Illumina HiSeq 2000 platform. Clean reads were further assembled de novo into a transcriptome dataset comprising of 514,747 unigenes with N50 length of 578 bp and were further aligned to the public databases. Genebank non-redundant (Nr), Viridiplantae, Gene Ontology (GO), KOG, and KEGG databases classification suggested enrichment of these unigenes in 30 GO categories, 26 KOG, and 127 pathways, respectively. Out of 265,158 genes that were differentially expressed in response to salt treatment, 134,566 and 130,592 genes were significantly up and down-regulated, respectively. Upon placing all the differentially expressed genes (DEG) in known signaling pathways, it was evident that most of the DEGs involved in cytokinin, ethylene, auxin, abscisic acid, gibberellin, and Ca^2+^ mediated signaling pathways were up-regulated. Furthermore, GO enrichment analysis was performed using REVIGO and up-regulation of multiple genes involved in various biological processes in *chilense* under salinity were identified. Through pathway analysis of DEGs, “Wnt signaling pathway” was identified as a novel pathway for the response to the salinity stress. Moreover, key genes for salinity tolerance, such as genes encoding proline and arginine metabolism, ROS scavenging system, transporters, osmotic regulation, defense and stress response, homeostasis and transcription factors were not only salt-induced but also showed higher expression in *S. chilense* as compared to *S. lycopersicum*. Thus indicating that these genes may have an important role in salinity tolerance in *S. chilense*. Overall, the results of this study improve our understanding on possible molecular mechanisms underlying salt tolerance in plants in general and tomato in particular.

## Introduction

Soil salinity is one of the major abiotic stresses that significantly affects the plant growth and crop yields. Salinity stress can have a major impact on the global food production^1,2^. To date, approximately 10% of the world’s total land area (950 Mha), 20% of the world’s aerable land (300 Mha), and 50% of the total irrigated land (230 Mha) are affected by soil salinization. Further, it is expected to influence 50% of total cultivated land in 2050 at a disquieting rate^3,4^. Every year almost 12 billion US$ are globally lost due to salt stress that significantly affects the agricultural production^5,6^. High soil salinity stress affects plants, mainly in three ways: (a) ionic stress arising from increased levels of toxic ions such as Na^+^ and Cl^−^ causing serious ionic imbalance, (b) osmotic stress due to the limited water availability leading to augmented osmotic pressure, and (c) oxidative damage, ultimately leading to the plant growth retardation and death^7–9^. Plants have to generate a series of significant salt-tolerance mechanisms to minimize the adverse effects of salt stress, and develop various physiological, biochemical, and molecular processes. This is acheived by regulating the expression of salt-responsive genes and other sophisticated strategies for proper membrane transport, ion transport, signal transduction, redox regulation, metabolic networks, transcription factors, amine and polyamine metabolism, oxidation–reduction, phyto-hormones, and reactive oxygen species (ROS) scavenging^10–12^.

Mechanisms for improved salt tolerance are well-recognized in *Arabidopsis* and other crops, which mainly comprise of calcium-dependent protein kinase (CDPK) pathway, salt overly sensitive (SOS) pathway, and mitogen-activated protein kinase (MAPK) pathway^13,14^. Plasma membrane Na^+^/H^+^ antiporter such as *SOS1* is involved in sodium exclusion from the cytosol^15^. SOS2 [Calcineurin B-like protein (CBL)-CBL-interacting protein kinase (CIPK)] is a serine/threonine-protein kinase which interacts with SOS3-calcium sensor (CBL) to regulate the activity of Na^+^/H^+^ exchanger *SOS1* by which these complexes confer salt resistance^15–17^. Interaction of CBL1 with CIPK24, CIPK25, and CIPK26 is known to regulate the Na^+^/K^+^ homeostasis^18^. Plasma membrane Na^+^/H^+^ antiporters (*SOS1)* that are generated in response to salt stress were also identified in *Populus euphratica*^19^. Additionally, several specific genes/gene families such as high-affinity K^+^ transporter (HKT) and Na^+^/H^+^ antiporters (NHX) conferring salinity tolerance have been reported in *Arabidopsis thaliana*^20^. Moreover, the contribution of cytochrome P450 genes in salt stress response has been established in the salt-tolerant tree species *Populus euphratica*^21^. Besides these, several transcription factors (TFs) belonging to *bZIP*, *MYB*, *bHLH*, *C2H2-Dof*, *AP2-EREBP*, and *WRKY* super families have been reported to play crucial role in imparting salt tolerance response in plants^22–24^.

Ca^2+^ plays a key role in plant adaptation against critical stress conditions and works as a second messenger in regulating growth and development of plants^25^. Ca^2+^ sensors are classified mainly into three families, comprising calcineurin B-like proteins, Ca^2+^-dependent protein kinases (CDPKs), and calmodulin^26–28^. Many transcription factors are directly activated by Ca^2+^/calmodulin, containing MYBs^29^, calmodulin binding transcription activators (CAMTAs)^30^ and GT-element-binding-like proteins^31^. Sulphur acclimatization also plays an essential role in the salt tolerance mechanism in plants through adaptation of metabolic modifications^32,33^. S-adenosyl methionine and arginine decarboxylase (ADC) are involved in polyamines (PAs) synthesis such as putrescine (Put) which is known to improve tolerance against salinity stress^34,35^.

Tomato (*Solanum lycopersicum* L*.*) is the second most consumed vegetable crop after potato^36^ and certainly the globally-widespread garden crop^37^. Tomato fruits are rich source of natural pigments such as lycopene, β-carotene, dietary fiber, vitamin A, vitamin E, potassium, and vitamin C. Lycopene, a pigment enriched with antioxidant properties is used for the therapeutic purpose to prevent cancer^38^. Most of the cultivated tomatoes are known to be highly sensitive to salinity at different stages of plant growth and fail to produce higher yield^37^. Therefore, it becomes necessary to identify the key salt responsive genes that confer salt tolerance and facilitate plant survival during high salinity^39^. Estimation by Miller and Tanksley^40^ suggested that cultivated tomato families within *S. lycopersicum* have less than five per cent of the total genetic variation. Therefore, genes involved in salt tolerance may not occur in the cultivated tomato species^37^. However, wild relatives of tomato such as *S. chilense*, *S. pimpinellifolium, S. peruvianum, S. hirsutum*, and *S. pennellii* are known to possess genes essential for improvement of abiotic stress tolerance.

However, till date, there is no sufficient information available about the salinity tolerance in *S. chilense*. Hence, it becomes more important to know stress-responsive mechanisms evolved by *S. chilense* that allows it to survive in life-threatening aridity, high temperature, drought, and salt stress environment in Northern Chile of Atacama Desert^41^. Earlier studies have suggested that *S. chilense* shows a promising response to drought and salt-tolerance as compared to the cultivated tomato^42–44^. Besides these, enhanced salt adaptation and tolerance activities such as mobilization of H_2_O_2_ components, higher plant water holding capacity, growth, and increased enzymatic antioxidant capacity are well-documented in *S. chilense*^42,44–46^. Unfortunately, the information on molecular salt stress mechanisms of this species during salinity is lacking. Therefore, it is imperative to elucidate gene expression profiles of this species under salt stress.

Capturing expression pattern of salt-responsive genes by high-throughput sequencing technologies makes it possible to resolve important questions realated to salinity stress tolerance^47,48^. RNASeq technology remains an essential tool for elucidating responsive mechanisms against salinity conditions in tomato. Furthermore, the assembly of de novo transcript sequences represents an efficient and cost-effective method to recognize salt responsive genes in tomato wild relative *S. chilense*. Transcriptome studies in plants with salinity stress have been documented in many crops such as canola^49^, common bean^50^, ice plant^51^, Kentucky bluegrass^52^, wheat^53^ and desert poplar^54^. In this article, we carried out a comparative transcriptome analysis of *S. lycopersicum* and a wild relative of tomato, *S. chilense* based on the high-throughput Illumina HiSeq 2000 platform to deliver a comprehensive molecular data for identification of molecular markers, genes and pathway, and biochemical, molecular, and physiological basis of salt tolerance in *S. chilense*. These findings will provide valuable information for breeding salt tolerance in tomatoes.

## Results and discussion

### An overview of the de novo assembly of *S. chilense* transcriptome

Illumina HiSeq 2000 platform was used for RNASeq. A total of 88.37 million reads were generated in *Chilense*_Control, 101.23 million in *Chilense*_Treated, 101.50 million in DVRT-1_Control, and 100.23 million in DVRT-1_Treated. Over 96% of the reads had Phred-like quality scores at the Q30 level (error < 0.1%) (Table S1). We obtained 87.08 million clean reads in *Chilense*_Control, 100.01 million in *Chilense*_Treated, 100.08 million in DVRT-1_Control, and 98.82 million in DVRT-1_Treated, with 44.97% average GC content (Table S1). Further, all clean reads with high-quality regions were assembled into 514,747 unigenes with a maximum size of 46,034 bp, a minimum unigene size of 300 bp, 591.57 bp mean length, and a 578 bp of N50 value (Table S2). The unigene length, random distribution, classification, and annotation are presented in Fig. S1 and Fig. S2a to Fig. S2d.

### Functional annotation and classification of transcriptome sequences

To perform the functional annotation of unigene dataset, we compared each unigene against the Genebank Nr, Viridiplantae, Gene Ontology (GO), euKaryotic Orthologous Groups (KOG), and Kyoto Encyclopedia of Genes and Genomes (KEGG) databases. Among the 514,747 unigenes, 201,803 (39.20%) were significantly annotated in the Genebank Nr database with the maximum hit rate against all four public databases (*E* value < 1.0 E−50). The annotated unigenes in Viridiplantae, GO, KOG, and KEGG databases were 160,452 (31%), 108,355 (21.05%), 126,937 (24%), and 7,923 (1.53%), respectively. Since the Whole Genome Sequence (WGS) of *S. chilense* was unavailable, annotation of unigenes was done by comparing the assembled transcriptome data to the public domain databases. Among the 514,747 assembled unigenes, 108,355 unigenes were assigned into 30 GO categories according to their sequence homology under three main categories, viz., cellular component (CC), biological process (BP) and molecular function (MF) each separately with 10 GO terms (Fig. S3). With respect to cellular components, integral component of membrane (41.18%) were the most dominant groups followed by nucleus (17.23%) and cytoplasm (11.94%) (Fig. S3). Among biological process, ATP binding process (31.23%), DNA binding (10.01%) and metal ion binding (9.82%) were highly represented (Fig. S3). Under molecular function term, transcription (28.82%), regulation of transcription (26.09%) and translation (20.85%) were most represented (Fig. S3). To further identify the biological pathways that were activated in *S. chilense*, 7,923 annotated unigenes were mapped to the reference canonical pathways in the KEGG and were assigned to 127 KEGG pathways. The most represented pathways were genetic information processing (56.39%), signaling and cellular processes (18.75%) and metabolism (17.56%) (Fig. S4 and Table S3). Furthermore, KOG classification indicated that 126,937 (24%) transcriptome sequences were gathered into 26 functional categories. Among the 25 groups, general function prediction represented the most enriched term (19.72%), which was followed by signal transduction mechanism (14.80%) and post-translational modification (Fig. S5 and Table S4).

### Identification of SSR

By screening 514,747 transcriptome sequences, a total of 106,239 potential SSRs were identified, which distributed among 81,256 unigenes, and including 18,593 unigenes with more than one SSR. Among them, 8,784 SSRs were compound SSRs. The most abundant SSR type was mono-nucleotide (65.53%), followed by di-nucleotide (8.52%), tri-nucleotide (6.70%), tetra-nucleotide (0.59%), penta-nucleotide (0.09%), and hexa-nucleotide (0.06%) (Fig. S6, Table S5, and Table S6).

### Identification of transcription factors families

**Figure 1:**
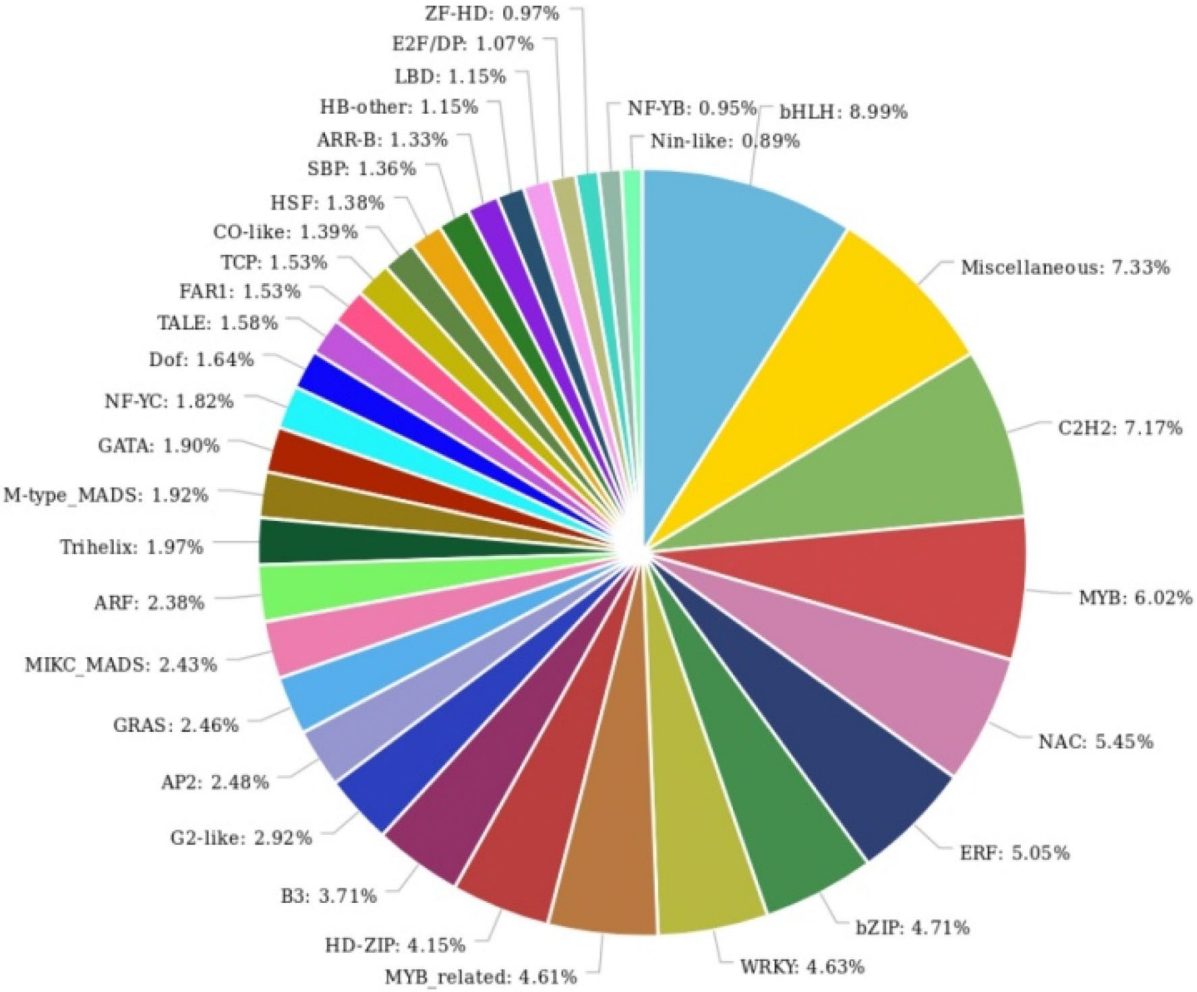
Identification of transcription factors families of assembled transcripts. Plant TFDB tool was used for identification of potential transcription factors families. A total of 57 transcription factor families, containing 6,353 unigenes were identified.

Transcription factors (TF) play a significant role in plant development processes and regulate the expression of specific genes under different stress conditions^55^. Plant TFDB online tool was used for identification of potential transcription factor families. A total of 57 transcription factor families, containing 6,353 unigenes were identified (Table S7). Out of these, *bHLH* (8.99%) was found to be the most abundant TF family, followed by *C2H2* (7.123%), *MYB* (6.02%), *NAC* (5.45%), *ERF* (5.05%), *bZIP* (4.71%), *WRKY* (4.63%), and *MYB*-related (4.61%) (Fig. 1).

### Analysis of DEGs

Differentially expressed gene (DEGs) analysis is an important approach to identify the salinity tolerance responsive genes in *S. chilense*. Therefore, DEGs were identified from the RNA-seq data based on the criteria of fold change ≥ 2 for the comparisons salinity *Chilense*_Treated versus *Chilense*_Control, DVRT-1_Treated versus DVRT-1_Control, *Chilense*_Treated versus DVRT-1_Treated and an FDR < 0.05 (Tables 1, 2, 3, 4, 5). In studying expression pattern of DEGs in *Chilense*_Treated versus *Chilense*_Control , total of 67,293 DEGs (30,924 up-regulated and 36,369 down-regulated genes) were identified (Fig. S7a, Table S8, and Table S9), while 81,882 DEGs (42,426 up-regulated and 39,456 down-regulated genes) presented noteworthy changes in DVRT-1_Treated versus DVRT-1_Control comparison group (Fig. S7b, Table S8, and Table S10). In *Chilense*_Treated versus DVRT-1_Treated comparison group, 1,15, 983 DEGs (61,216 genes up-regulated and 54,767 down-regulated genes) were identified (Fig. S7c, Table S8, and Table S11). Heat map and cluster analysis revealed that the number of genes up-regulated under salinity in both *S. chilense* as well as DVRT-1 was higher compared to the down-regulated genes (Fig. S8).

**Table 1:** List of putative candidate genes related with amine and polyamine metabolism, oxidation–reduction and hormone related for salt tolerance in *S. Chilense*.

Type	Unigene ID	*Chilense*_Treated versus DVRT-1_Treated (log2FC)	*Chilense*_Treated versus *Chilense*_Control (log2FC)	DVRT-1_Treated versus DVRT-1_Control (log2FC*)*	*p*-value	E-value	Gene description
**Amine and polyamine biosynthesis and metabolism**							
	Unigene_209588	8.32	6.714	6.48	0.014508	1.70E−230	Polyamine oxidase 1 isoform X1 [GO:0016491]
	Unigene_124485	7.42	6.52	4.25	0.021411	2.00E−249	Polyamine oxidase [GO:0046592]
	Unigene_160311 **(Q1)**	4.25	3.132	2.1	0.141211	2.00E−25	Arginine decarboxylase [GO:0008792](EC 4.1.1.19)
	Unigene_210393	2.25	3.193	2.78	0.37862	7.00E−72	Polyamine biosynthetic process [GO:0006596]
	Unigene_278216 **(Q2)**	2.81	3.856	1.25	0.383927	1.00E−33	betaine-aldehyde dehydrogenase [GO:0008802]; choline dehydrogenase [GO:0008812] (EC 1.1.99.1)
	Unigene_191720 **(Q3)**	3.04	2.778	2.32	0.52688	1.70E−10	Pyrroline-5-carboxylate reductase (EC 1.5.1.2)[GO:0004735], L-proline biosynthetic process[GO:0055129],
	Unigene_22288 **(Q4)**	4.38	4.78	2.14	0.124408	6.40E−202	Trehalose 6-phosphate phosphatase (EC 3.1.3.12) [GO:0004805]
**Oxidation–reduction**							
	Unigene_202051 **(Q5)**	10.26	5.6	2.05	0.000142	4.40E−270	Cytochrome P450 [GO:0004497]
	Unigene_213820	6.09	2.2	1.45	0.026086	2.20E−227	Cytochrome P450 71 family protein ]GO:0005506]
	Unigene_171236	6.09	4.15	2.13	0.058693	8.60E−21	Cytochrome P450 89A2-like [GO:0016705]
	Unigene_201771	5.12	3.54	1.78	0.067246	8.50E−44	Cytochrome P450 83B1-like [GO:0020037]
	Unigene_144908 **(Q6)**	4.91	3.46	1.21	0.088437	8.80E−103	Cytochrome P450-type monooxygenase 97C11 [GO:0016123]
	Unigene_22157	4.82	3.33	2.85	0.062834	2.50E−11	Cytochrome P450-type monooxygenase 97A29 [GO:0010291]
	Unigene_182664	10.22	6.714	3.36	0.004673	1.30E−123	Oxidoreductase activity [GO:0016491]
	Unigene_112135 **(Q7)**	3.15	2.58	2.07	0.232691	4.00E−146	L-ascorbate oxidase [GO:0005507]
**Hormone related**							
	Unigene_143855 **(Q8)**	3.32	2.49	1.22	0.263322	8.80E−31	12-oxophytodienoate reductase 1(EC 1.3.1.42) Lipid metabolism; oxylipin biosynthesis.[GO:0010181]
	Unigene_58417 **(Q9)**	3.66	2.12	1.45	0.183994	2.10E−39	1-aminocyclopropane-1-carboxylate oxidase homolog (Protein E8)[GO:0009693]
	Unigene_202800	2.81	2.6	1.14	0.383927	9.20E−37	1-aminocyclopropane-1-carboxylate deaminase activity [GO:0008660]
	Unigene_4025 **(Q10)**	4.16	4.11	3.27	0.140564	6.20E−166	Adenylate isopentenyltransferase (Isopentenyltransferase 1)[GO:0005634]
	Unigene_193865	5.82	4.25	2.96	0.045743	6.20E−128	Auxin response factor [GO:0003677]
	Unigene_19919	3.32	2.88	1.36	0.415692	1.80E−40	Cytokinin hydroxylase-like [GO:0005506]
	Unigene_182310	7.22	4.53	4.82	0.010413	1.30E−293	Cytokinin oxidase/dehydrogenase-like [GO:0009690]
	Unigene_62211 **(Q11)**	4.29	2.99	2.01	0.126544	7.10E−26	Cytokinin riboside 5′-monophosphate phosphoribohydrolase (EC 3.2.2.n1)[GO:0005634]
	Unigene_18638 **(Q12)**	5.04	3.22	1.54	0.070267	3.20E−226	Gibberellin 20-oxidase-1[GO:0009416]
	Unigene_207701	2.25	4.51	1.11	0.373985	3.70E−219	Gibberellin 20-oxidase-3[GO:0009416]
	Unigene_104465	7.19	3.15	2.63	0.025356	3.50E−191	Linoleate 9S-lipoxygenase A (EC 1.13.11.58) (Lipoxygenase A)[GO:0005737]
	Unigene_120281 **(Q13 A & B)**	4.6	4.14	3.06	0.046356	4.80E−32	Lipoxygenase (EC 1.13.11.-)[GO:0016702]
	Unigene_340892	2.58	2.19	1.45	0.447289	0.000019	Lipoxygenase homology domain-containing protein 1-like (Fragment)[GO:0004096]
	Unigene_29074 **(Q14)**	3.7	3.22	2.15	0.175302	1.70E−24	allene oxide synthase 2-like [GO:0004497]
	Unigene_174090	5.52	4.15	2.43	0.066435	4.80E−48	Putative auxin-induced protein 15A-like[GO:0009733]
	Unigene_108814	7.17	6.47	2.04	0.022346	4.80E−32	response to abscisic acid [GO:0009737]
	Unigene_131016	4.21	3.81	1.55	0.144589	8.50E−24	auxin catabolic process [GO:0009852]
	Unigene_27210	7.7	3.75	3.11	0.017156	3.90E−29	response to auxin [GO:0009733]

These candidate genes were up-regulated in both the *Chilense*_Treated versus DVRT-1_Treated and *Chilense*_Treated versus *Chilense*_Control comparison groups compare to DVRT-1_Treated versus DVRT-1_Control groups with categorized according to predicted gene function (corrected *p* value ˂ 0.05). Q1 to Q14 are highlighted bold in brackets shows gene expression was confirmed by qPCR.

**Table 2:** List of putative candidate genes related with ROS scavenging and signaling transduction for salt tolerance in *S. Chilense*.

Type	Unigene ID	*Chilense*_Treated versus DVRT-1_Treated (log2FC)	*Chilense*_Treated versus *Chilense*_Control (log2FC)	DVRT*-1*_Treated versus DVRT-1_Control (log2FC)	*p*-value	E-value	Gene description
**ROS scavenging**							
	Unigene_1506 **(Q15 A & B)**	5.42	3.068	2.5	0.027109	1.90E−115	Superoxide dismutase (EC 1.15.1.1) [GO:0004784]
	Unigene_293323 **(Q16)**	5.32	4.16	2.608	0.076862	5.20E−246	Catalase (EC 1.11.1.6)[GO:0004096]
	Unigene_187400 **(Q17)**	5.97	3.99	2.416	0.057172	1.40E−137	Peroxidase (EC 1.11.1.7)[GO:0004601]
	Unigene_207937 **(Q18)**	5.74	3.05	2.416	0.020226	1.50E−115	Glutathione peroxidase activity [GO:0004602]
	Unigene_149763 **(Q19)**	5.42	2.416	1.54	0.048036	2.20E−65	Glutaredoxin [GO:0055114]
	Unigene_160061 **(Q20)**	6.41	5.21	2.252	0.036423	1.60E−192	Thioredoxin reductase (EC 1.8.1.9) [GO:0004791]
	Unigene_53734	2.85	2.6	1.14	0.24568	2.60E−30	Response to singlet oxygen [GO:0000304]
	Unigene_92698	3.37	3.696	2.05	0.21658	5.50E−67	Response to hydrogen peroxide [GO:0042542]
	Unigene_145695	3.17	3.778	2.66	0.237707	0.000049	Removal of superoxide radicals [GO:0019430]
	Unigene_92623 **(Q21)**	3.59	2.5	1.22	0.15875	0.00E+00	Glutathione synthetase (GSH-S) (EC 6.3.2.3) [GO:0000287]
**Signaling transduction**							
	Unigene_214194	8.81	4.178	2.54	0.009834	7.10E−252	Signal transduction [GO:0007165]
	Unigene_91400	8.07	4.132	2.02	0.007849	2.20E−82	Intracellular signal transduction [GO:0035556]
	Unigene_200805 **(Q22)**	9.24	2.77	2.46	0.010728	1.20E−168	Folylpolyglutamate synthase (EC 6.3.2.17) (Folylpoly-gamma-glutamate synthetase) (Tetrahydrofolylpolyglutamate synthase) [GO:0004326]
	Unigene_106494	8.45	4.55	3.12	0.007182	7.70E−110	Putative calcium-binding EF hand family protein-like [GO:0005509]
	Unigene_92461	7.49	6.59	2.95	0.030527	3.70E−27	Protein serine/threonine kinase activity [GO:0004674]
	Unigene_144328	5.41	5.11	4.55	0.073663	4.70E−63	Putative LRR receptor-like serine/threonine-protein kinase-like [GO:0004672]
	Unigene_203648 **(Q23)**	5.36	2.77	1.47	0.089392	3.70E−30	Imidazoleglycerol-phosphate dehydratase (EC 4.2.1.19) [GO:0000105] histidine biosynthetic process
	Unigene_186700	4.04	2.78	2.14	0.160375	2.00E−41	Urocanate hydratase (Urocanase) (EC 4.2.1.49) (Imidazolonepropionate hydrolase) [GO:0016153] histidine catabolic process
	Unigene_218320 **(Q24)**	3.99	2.88	1.25	0.149808	1.20E−37	Casein kinase II subunit beta (CK II beta) [GO:0005956] protein kinase regulator activity
	Unigene_158102	5.48	4.85	2.14	0.071379	8.70E−30	Putative casein kinase I-like [GO:0004672] protein kinase activity
	Unigene_123805 **(Q25)**	3.53	3.22	2.64	0.152543	6.80E−162	Phosphoenolpyruvate carboxylase kinase 1 [GO:0004683] calcium-dependent protein serine/threonine kinase activity
	Unigene_134889 **(Q26)**	2.4	4.14	1.57	0.310471	5.70E−132	Calcineurin B-like 10 (Calcineurin B-like molecule) [GO:0005509] calcium ion binding
	Unigene_128888	3.32	3.11	1.44	0.263322	3.10E−35	Calcineurin subunit B [GO:0005509] calcium ion binding
	Unigene_183215 **(Q27)**	3.18	2.81	2.15	0.23838	4.60E−98	receptor-like protein kinase HERK 1 [GO:0004672] protein kinase activity
	Unigene_6117	2.95	2.33	2.05	0.270844	8.00E−64	Putative histidine kinase 2-like [GO:0000155] phosphorelay sensor kinase activity
	Unigene_109301 **(Q28)**	2.81	3.22	2.58	0.281171	1.40E−33	14-3-3 protein zeta [GO:0019904]
	Unigene_106494	8.45	4.14	2.16	0.007182	7.70E−110	Calcium ion binding [GO:0005509]
	Unigene_218717 **(Q29 A & B)**	6.01	4.44	2.25	0.022576	4.80E−36	Calmodulin [GO:0005509] calcium ion binding
	Unigene_159681 **(Q30)**	5.84	6.25	5.64	0.182702	4.60E−25	MLO-like protein [GO:0005516]calmodulin binding
	Unigene_40787	3.09	3.11	2.46	0.239339	6.80E−99	Calmodulin-binding transcription factor SR1L [GO:0003677]
	Unigene_163043 **(Q31)**	3.52	2.22	1.45	0.227879	1.60E−09	Calcium/calmodulin-dependent protein kinase (CaM kinase) II [GO:0004683]
	Unigene_70899	3.45	2.14	1.74	0.205694	8.30E−236	Calmodulin-binding transcription factor SR4 [GO:0003677]
	Unigene_144565 **(Q32)**	3.39	3.65	1.11	0.250259	1.10E−22	Methionine synthase (EC 2.1.1.13) (5-methyltetrahydrofolate-homocysteine methyltransferase)[GO:0008705]
	Unigene_83655 **(Q33)**	2.65	2.55	1.17	0.217126	2.60E−153	S-adenosylmethionine synthase 2 (AdoMet synthase 2) (EC 2.5.1.6) (Methionine adenosyltransferase 2) (MAT 2) [GO:0004478]
	Unigene_224888 **(Q34)**	2.57	3.21	1.55	0.330009	1.50E−55	Serine hydroxymethyltransferase 1, mitochondrial (AtSHMT1) (EC 2.1.2.1) [GO:0004372]
	Unigene_9629	3	2.55	1.23	0.264096	8.20E−130	Phosphotransferase (EC 2.7.1.-) hexokinase activity [GO:0004396]

These candidate genes were up-regulated in both the *Chilense*_Treated versus DVRT-1_Treated and *Chilense*_Treated versus *Chilense*_Control comparison groups compare to DVRT-1_Treated versus DVRT-1_Control groups with categorized according to predicted gene function (corrected *p* value ˂ 0.05). Q15 to Q34 are highlighted bold in brackets shows gene expression was confirmed by qPCR.

**Table 3:** List of putative candidate genes related with transporters for salt tolerance in *S. Chilense*.

Type	Unigene ID	*Chilense*_Treated versus DVRT-1_Treated (log2FC)	*Chilense*_Treated versus *Chilense*_Control (log2FC)	DVRT-1_Treated versus DVRT-1_Control (log2FC)	*p*-value	E-value	Gene description
**Transporters**							
	Unigene_177780	8.02	4.78	2.2	0.003759	2.10E−38	ATPase activity [GO:0016887]
	Unigene_293030 **(Q35)**	3.87	3.41	2.63	0.163347	8.80E−120	V-type proton ATPase subunit a [GO:0000220]
	Unigene_150283 **(Q36)**	3.62	3.11	2.32	0.185275	2.20E−53	Vacuolar cation/proton exchanger [GO:0015369]
	Unigene_176401	4.17	3.193	2.14	0.148174	7.70E−113	Proton-transporting V-type ATPase, V1 domain [GO:0033180]
	Unigene_184700	4.04	3.193	2.11	0.160375	2.10E−38	Cation-transporting ATPase activity [GO:0019829]
	Unigene_192446	3.17	2.69	2.27	0.251291	4.30E−16	Sugar abc transporter, putative (EC 3.6.3.17) (Fragment) [GO:0005524]
	Unigene_127043	3	2.22	1.31	0.333765	0.000000021	Sodium/potassium-transporting ATPase subunit alpha [GO:0005391]
	Unigene_149961 **(Q37)**	4.47	3.25	1.11	0.110557	5.30E−10	Sodium/hydrogen exchanger 8-like isoform X1 [GO:0015299]
	Unigene_85698 (**Q38 A & B**)	3.92	2.58	1.14	0.15895	1.20E−48	Sodium/hydrogen exchanger [GO:0005774]
	Unigene_34069	3.66	3.22	1.47	0.157855	1.60E−176	Sodium/hydrogen exchanger [GO:0005886]
	Unigene_34069	3.66	4.22	1.77	0.157855	1.60E−176	Calcium/hydrogen exchanger [GO:0005774]
	Unigene_124188	4.06	2.88	1.41	0.094613	1.60E−79	Putative V-type proton ATPase subunit c''-like [GO:0015078]
	Unigene_157402 **(Q39)**	4	3.55	2.14	0.100089	3.70E−145	Putative cyclic nucleotide-gated ion channel 4-like [GO:0005216]
	Unigene_40330	2.22	3.54	1.1	0.39256	1.10E−41	Putative cyclic nucleotide-gated channel 16-like [GO:0005216]
	Unigene_114086 **(Q40)**	3.12	2.55	1.14	0.237432	4.90E−113	Putative ABC transporter F family member 4-like [GO:0005634]
	Unigene_25096	4.18	3.22	1.14	0.09637	4.40E−257	Putative ABC transporter B family member 28-like [GO:0005524]
	Unigene_161663	3.81	2.22	1.88	0.288062	5.30E−10	Putative ABC transporter ATP-binding protein (EC 3.6.3.41) [GO:0005524]
	Unigene_137990 **(Q41)**	3.46	3.05	2.07	0.238503	2.20E−17	Plasma membrane ATPase 4 [GO:0005391]
	Unigene_290335 **(Q42)**	4.75	3.05	2.05	0.13336	1.30E−82	Plasma membrane H+-ATPase (EC 3.6.3.6) [GO:0005524]
	Unigene_181319	3.17	2.85	1.14	0.294357	1.70E−13	P-ATPase family transporter: copper ion heavy metal transporting P-type ATPase [GO:0000166]
	Unigene_63274	2.58	2.23	2.05	0.33884	0.00000043	Cation-transporting P-type ATPase [GO:0016021]
	Unigene_314042 **(Q43)**	4.09	4.03	2.2	0.156042	1.00E−18	Calcium-transporting ATPase 1 (EC 3.6.3.8) [GO:0005388]
	Unigene_346253	2.81	1.85	1.11	0.295604	1.90E−15	Ca2+ transporting ATPase [GO:0005391]
	Unigene_39994	3.81	3.55	2.12	0.187337	6.90E−26	ATP binding cassette (Abc) transporter, putative (EC 3.6.3.30) [GO:0005524]
	Unigene_120164	3.17	3.14	2.12	0.294357	0.00000056	Anion exchanger family [GO:0005452]
	Unigene_194760	5.97	4.44	2.14	0.054121	8.80E−19	ABC transporter I family member 11, chloroplastic-like isoform X1 [GO:0016887]
	Unigene_167776 **(Q44)**	3.17	2.85	1.36	0.294357	7.10E−11	ABC transporter B family member 25 [GO:0004672]
	Unigene_123443 **(Q45)**	3.91	2.15	1.23	0.175335	2.60E−12	ABC transporter B family member 2 [GO:0042626]
	Unigene_266471	2.58	2.51	1.11	0.447289	3.60E−25	ABC transporter B family [GO:0042626]
	Unigene_191339 **(Q46)**	3	2.19	1.11	0.333765	0.0000048	ABC transporter A family member 3 [GO:0016021]
	Unigene_134132	2.81	2.22	1.42	0.383927	0.00000003	ABC transporter [GO:0005524]
	Unigene_123913	5.96	3.93	2.19	0.031188	2.50E−51	Vacuolar transport [GO:0007034]
	Unigene_22215	8.12	3.856	2.27	0.012051	5.30E−128	transmembrane transport [GO:0055085]
	Unigene_175214	4.47	4.223	2.57	0.084489	1.30E−96	Vesicle-mediated transport [GO:0016192]
	Unigene_78131	4.39	2.778	2.58	0.051581	7.40E−12	Proton transmembrane transport [GO:1902600]
	Unigene_168052 **(Q47)**	4.32	2.193	2.72	0.135	4.00E−23	Potassium ion transport (Sodium/potassium)/proton exchanger 4 nhx4 [GO:0006813]
	Unigene_57528	6.34	6.001	2.38	0.047798	6.80E−70	Potassium ion transmembrane transport [GO:0071805]
	Unigene_128174	6.38	3.818	2.05	0.046923	3.50E−37	Regulation of ion transmembrane transport [GO:0034765]
	Unigene_297146 **(Q48)**	5.55	4.88	2.14	0.068805	1.90E−178	HKT1,2 (Na+ transporter) [GO:0008324]

These candidate genes were up-regulated in both the *Chilense*_Treated versus DVRT-1_Treated and *Chilense*_Treated versus *Chilense*_Control comparison groups compare to DVRT-1_Treated versus DVRT-1_Control groups with categorized according to predicted gene function (corrected *p* value ˂ 0.05). Q35 to Q48 are highlighted bold in brackets shows gene expression was confirmed by qPCR.

**Table 4:** List of putative candidate genes related with osmotic regulation, defence and stress and homeostasis for salt tolerance in *S. Chilense*.

Type	Unigene ID	*Chilense*_Treated versus DVRT-1_Treated (log2FC)	*Chilense*_Treated versus *Chilense*_Control (log2FC)	DVRT-1_Treated versus DVRT-1_Control (log2FC*)*	*p*-value	E-value	Gene description
**Osmotic regulation**							
	Unigene_167858 **(Q49)**	6.48	2.93	2.43	0.03133	1.20E−118	Beta-galactosidase (EC 3.2.1.23) [GO:0004565]
	Unigene_214160	4.32	3.21	1.44	0.135	3.10E−105	Annexin [GO:0005509]
	Unigene_156409 **(Q50)**	2.79	2.25	1.11	0.224345	3.80E−166	Putative annexin D4-like [GO:0005509]
	Unigene_114832 **(Q51)**	2.63	2.58	1.41	0.305681	2.80E−25	Alpha,alpha-trehalose-phosphate synthase [udp-forming] 1 [GO:0003824]
	Unigene_207284 **(Q52)**	9.06	4.21	3.16	0.000628	9.70E−289	PE1 pectinesterase 1(EC 3.1.1.11)Glycan metabolism [GO:0005576]
	Unigene_218862	6.68	3.25	2.11	0.156042	4.00E−24	Pectinesterase [GO:0005618]
**Defence and stress**							
	Unigene_62524	3.81	2.55	1.22	0.288062	0.000000027	Heat shock protein, mitochondrial [GO:0005737]
	Unigene_176752	4.46	4.11	3.27	0.118923	1.30E−224	Heat shock protein HSS1 [GO:0005524]
	Unigene_12837	3.46	3.32	2.14	0.238503	8.50E−54	Heat shock protein cognate 4 (Fragment) [GO:0005524]
	Unigene_172033 **(Q53)**	4.32	4.14	2.25	0.128087	1.00E−194	Heat shock protein 83 (Fragment) [GO:0006950]
	Unigene_185483 **(Q54)**	3.7	2.85	1.41	0.201447	3.10E−41	Heat shock protein 70 family [GO:0005524]
	Unigene_479285 **(Q55)**	2.72	2.85	1.11	0.207369	1.80E−36	Heat shock protein HSP 21 (Lycopersicum esculentum mRNA sequence) [GO:0006950]
	Unigene_7787 **(Q56)**	3.88	3.55	2.1	0.147214	1.40E−69	Heat shock factor protein HSF8 (Heat shock transcription factor 8) (HSTF 8) [GO:0003700]
	Unigene_129669	5.33	4.55	2.78	0.075029	1.00E−164	Heat shock factor protein HSF30 (Heat shock transcription factor 30) (HSTF 30) [GO:0043565]
	Unigene_152530	8.45	3.05	2.414	0.002971	2.80E−116	response to oxidative stress [GO:0006979]
	Unigene_209761	6.38	4.647	4.24	0.025575	4.60E−56	Response to salt stress [GO:0009651]
	Unigene_35066	13.49	6.235	4.73	1.22E−06	1.40E−41	Response to wounding [GO:0009611]
	Unigene_102040	7.27	5.981	4.51	0.02199	3.50E−123	Response to water deprivation [GO:0009414]
	Unigene_95307	7.08	7.732	4.83	0.021535	1.60E−59	Response to stress [GO:0006950]
	Unigene_217566	2.68	2.511	2.4	0.312641	1.00E−83	Hyperosmotic salinity response [GO:0042538]
	Unigene_56668	7.63	6.727	2.05	0.023712	2.20E−66	Defense response [GO:0006952]
	Unigene_94600	10.71	4.734	2.75	0.000195	5.40E−227	Secondary metabolite biosynthetic process [GO:0044550]
	Unigene_152266	8.07	3.193	2.99	0.010283	5.20E−233	Regulation of systemic acquired resistance [GO:0010112]
**Homeostasis**							
	Unigene_85698	3.92	3.001	2.13	0.15895	1.20E−48	Potassium ion homeostasis [GO:0055075]
	Unigene_79672	6.97	2.608	2.04	0.012902	1.60E−49	Iron ion homeostasis [GO:0055072]
	Unigene_75938	4.68	4.193	2.1	0.067834	8.70E−99	Calcium ion homeostasis [GO:0055074]
	Unigene_209051	8.95	6.562	2.46	0.00328	5.10E−77	Cellular transition metal ion homeostasis [GO:0046916]
	Unigene_138300 **(Q57)**	9.69	3.87	2.608	0.002576	5.60E−43	Ferritin (EC 1.16.3.1),cellular iron ion homeostasis [GO:0006879]
	Unigene_81510	4.74	3.22	2.01	0.088983	4.90E−111	Cell volume homeostasis [GO:0006884]
	Unigene_210709	10.61	6.771	2.48	0.000885	1.60E−34	Cell redox homeostasis [GO:0045454]

These candidate genes were up-regulated in both the *Chilense*_Treated versus DVRT-1_Treated and *Chilense*_Treated versus *Chilense*_Control comparison groups compare to DVRT-1_Treated versus DVRT-1_Control groups with categorized according to predicted gene function (corrected *p* value ˂ 0.05). Q49 to Q57 are highlighted bold in brackets shows gene expression was confirmed by qPCR.

**Table 5:** List of putative candidate genes related with transcription factor for salt tolerance in *S. Chilense*.

Type	Unigene ID	*Chilense*_Treated versus DVRT-1_Treated (log2FC)	*Chilense*_Treated versus *Chilense*_Control (log2FC)	DVRT-1_Treated versus DVRT-1_Control (log2FC*)*	*p*-value	E-value	Gene description
**Transcription factor**							
	Unigene_145610 **(Q58)**	3.38	2.06	1.25	0.212504	6.20E−62	Zinc finger SWIM domain-containing protein 7 [GO:0008270]
	Unigene_183317	4.25	2.92	2.035	0.141211	4.90E−26	Zinc finger protein 551 [GO:0003676]
	Unigene_167167	4.29	4.06	2.11	0.13802	7.80E−11	Zinc finger protein [GO:0003676]
	Unigene_150166 **(Q59)**	3.32	2.9	2.9	0.21741	0.0000014	Zinc finger CCCH domain-containing protein 1 [GO:0046872]
	Unigene_140108	3.25	3.25	2.51	0.207345	7.10E−119	Dof zinc finger protein [GO:0006355]
	Unigene_74574	3.7	3.22	1.14	0.201447	8.10E−41	Zinc finger BED domain-containing protein RICESLEEPER 3-like [GO:0046983]
	Unigene_286371	3.17	3.11	2.14	0.294357	0.0000005	Zinc finger (C3HC4-type RING finger) family protein isoform 1 [GO:0008270]
	Unigene_121135	2.58	2.52	1.85	0.447289	0.000000026	C2H2 zinc finger protein [GO:0008270]
	Unigene_202917	3.09	2.88	1.36	0.249633	5.80E−19	PREDICTED: zinc finger protein 347-like [GO:0003676]
	Unigene_186710	4.32	4.41	2.78	0.135	4.90E−19	PREDICTED: similar to Zinc finger protein 271 (Zinc finger protein 7) (HZF7) (Zinc finger protein ZNFphex133) [GO:000367]
	Unigene_27379 **(Q60)**	2.46	2.25	1.14	0.366114	0.0000001	WRKY72 [GO:0003700]
	Unigene_94493 **(Q61)**	4.88	4.14	2.11	0.045286	1.00E−100	WRKY3 [GO:0005634]
	Unigene_125785 **(Q62)**	3.58	3.36	1.14	0.183011	1.90E−17	WRKY1 [GO:0006351]
	Unigene_191009	4.98	3.65	2.52	0.039966	9.40E−56	WRKY transcription factor [GO:0043565]
	Unigene_182702	5.75	3.34	2.27	0.062242	2.30E−83	Putative WRKY transcription factor 57-like [GO:0006351]
	Unigene_116473	3.7	2.12	1.41	0.201447	9.20E−14	Putative WRKY transcription factor 2-like [GO:0043565]
	Unigene_7186	5.18	4.11	2.22	0.038646	1.20E−175	Putative transcription factor bHLH-like [GO:0046983]
	Unigene_213998 **(Q63)**	3.64	2.11	1.14	0.182406	4.50E−09	Transcription factor bHLH96 [GO:0046983]
	Unigene_127217	2.57	2.66	1.11	0.321855	3.30E−89	Transcription factor bHLH79 [GO:0046983]
	Unigene_167842	5.92	4.47	1.58	0.058392	1.10E−61	Transcription factor bHLH69-like isoform X1 [GO:0046983]
	Unigene_158779	6.42	5.25	2.22	0.045866	1.70E−80	TCP transcription factor 23 [GO:0003677]
	Unigene_288987	5.24	4.73	3.22	0.059725	2.30E−53	R3 MYB transcription factor (Fragment) [GO:0003677]
	Unigene_121825	4.67	3.96	2.55	0.106379	2.70E−32	Putative myb-related protein A-like [GO:0003677]
	Unigene_126646	6.13	5.22	3.21	0.033263	1.20E−48	Transcription factor MYB59 [GO:0003677]
	Unigene_186678 **(Q64)**	2.91	3.42	2.22	0.276721	8.20E−35	Putative transcription factor MYB48-like [GO:0003677]
	Unigene_196370	2.42	2.24	1.14	0.412718	2.60E−35	MYBR1 [GO:0003677]
	Unigene_7447	4.39	3.69	2.14	0.119896	6.30E−100	MYB-like transcriptional factor MYB76 [GO:0001135]
	Unigene_91708 **(Q65)**	3	3.11	2.27	0.263046	9.90E−45	Myb 12 transcription factor [GO:0003677]
	Unigene_90410	7.04	5.99	3.11	0.02823	6.00E−18	Putative transcription factor ICE1-like [GO:0046983]
	Unigene_1208	3.1	2.88	2.14	0.24601	1.10E−79	Putative bZIP transcription factor family protein 2-like [GO:0003700]
	Unigene_120501	4.53	3.63	1.25	0.062942	1.20E−58	BZIP transcription factor [GO:0003700]
	Unigene_134802	6.68	3.17	2.22	0.019254	7.80E-−179	NAC2-domain containing protein [GO:0003677]
	Unigene_207656	5.53	7.05	3.22	0.038833	2.70E−177	NAC transcription factor (Sinor-like protein 1) [GO:0005634]
	Unigene_171741	8.73	7.08	7.03	0.010463	8.40E−52	NAC domain-containing protein 104-like isoform X1 (NAC domain-containing protein 104-like isoform X2) [GO:0006355]
	Unigene_118464	4.09	4.09	3.28	0.09816	2.30E−152	Putative NAC transcription factor 29-like [GO:0006355]
	Unigene_2437	4.43	3.36	2.21	0.126832	3.10E−32	Putative GATA transcription factor 24-like [GO:0008270]
	Unigene_152860 **(Q66)**	2.17	2.31	1.14	0.437212	4.30E−17	GATA transcription factor 24 [GO:0005634]
	Unigene_217830	3.91	2.7	2.84	0.128183	9.00E−67	GATA transcription factor [GO:0003682]
	Unigene_212668 **(Q67)**	4.86	3.36	2.11	0.057326	1.10E−82	Ethylene response factor 4 [GO:0005634]
	Unigene_63022 **(Q68)**	3.71	2.27	1.41	0.14701	9.30E−236	Auxin response factor 2B (SlARF2B) [GO:0005634]
	Unigene_103235 **(Q69)**	6.01	4.55	2.25	0.01963	6.00E−155	Auxin response factor 2A (SlARF2A) [GO:0006351]
	Unigene_52365	3.4	2.6	1.25	0.212806	1.70E−236	Auxin response factor [GO:0006355]
	Unigene_198879	3.29	2.33	1.11	0.222099	1.10E−17	AP2 transcription factor (Fragment) [GO:0007275]

These candidate genes were up-regulated in both the *Chilense*_Treated versus DVRT-1_Treated and *Chilense*_Treated versus *Chilense*_Control comparison groups compare to DVRT-1_Treated versus DVRT-1_Control groups with categorized according to predicted gene function (corrected *p* value ˂ 0.05). Q58 to Q69 are highlighted bold in brackets shows gene expression was confirmed by qPCR.

### Functional categorization of salinity-treated *S. chilense* and DVRT-1 DEGs

Blast2GO was used for GO term enrichment analysis for further understanding the biological function of the DEGs. The identified DEGs were highly abundant in 27 GO terms (*p* < 0.05), which showed that these DEGs were highly expressed in cellular processes in response to a stimulus metabolic process, localization, biological regulation and protein containing-complex (Fig. S9). Most of the upregulated DEGs under salt stress were abundant in catalytic activity, transcription regulator and molecular transducer. Whereas all the down-regulated DEGs were involved in binding, molecular function regulator and catalytic activity (Fig. S9). GO enrichment pathway analyses of these DEGs was conducted for pathway prediction. It was revealed that the DEGs were overrepresented in pathway terms [*Wnt* signaling (“Wingless-related integration site”), Epidermal growth factor (EGF) receptor signaling, Platelet-derived growth factor (PDGF) signaling], de novo purine biosynthesis, and ascorbate degradation (Fig. S10). Among these pathways, the “Wnt signaling pathway” was over-represented and has not been reported previously with regard to the salinity function. Nevertheless, presence of *Wnt* or *Wnt*-like signaling pathway in plants system is still unknown. Armadillo repeat proteins (ARM) play an important role in various developmental and stress signaling pathways in plants such as rice (*Oryza sativa* L.)^56^, *Arabidopsis*^57^, cotton (*Gossypium hirsutum*)^58^, and *Physcomitrella patens*^59^. Functional characteristics of ARM are similar to β-catenin, the key transcriptional modulator of *Wnt* signaling and *Wnt*-like signaling pathway in metazoan. For instance, in *Arabidopsis* SHAGGY-related protein kinase (ASK), 70% is similar to glycogen synthase kinase-3(GSK3) from mammals, the principal modulator of diverged biological functions in both plants and animals^60^. In plants, mostly GSKs participate in salt stress response and brassinosteroid signaling^61^. In consequence of these statements, SHAGGY-related protein kinase (ASK) and GSK3 were found to have higher expressions displayed in *Chilense*_Treated versus DVRT-1_Treated, which are the key genes responsible for the *Wnt* signaling (Table S9, S10 and S11). Therefore, our results suggest that *Wnt* signaling plays a crucial role in imparting salt tolerance in *S. chilense*. Pathway analyses of unigenes revealed that protein ubiquitination pathway was over-represented followed by glycan metabolism and carbohydrate degradation (Table S12, Fig. S11a to Fig. S11d).

**Figure 2:**
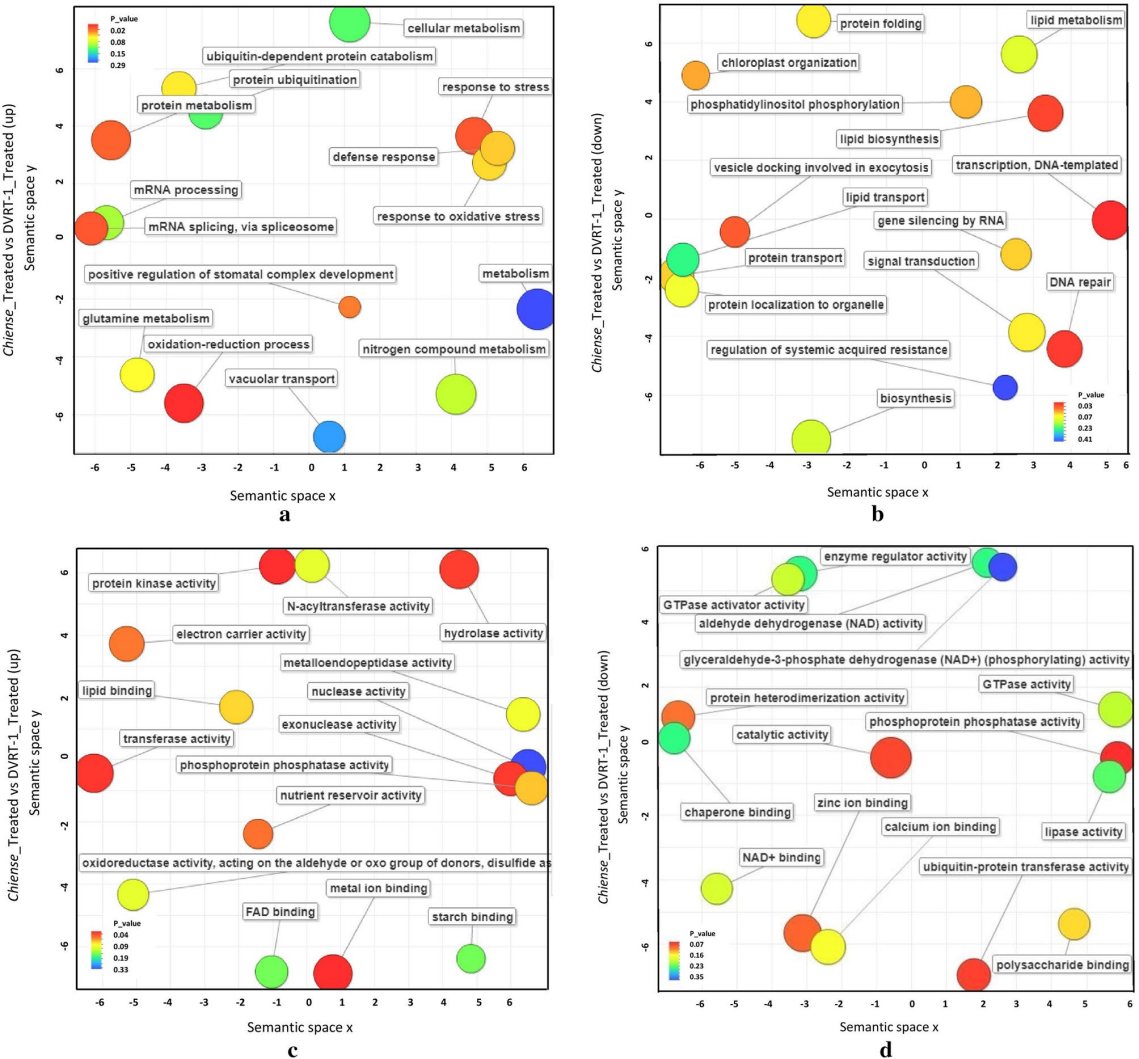
Gene Ontology (GO) enrichment analysis of differentially expressed genes using REVIGO. The top fifteen GO terms enriched in *Chilense*_Treated_vs_DVRT-1_Treated DEGs, determined based on the lowest p values, were analyzed by REVIGO. (**a,b**) The enriched GO terms from the REVIGO analysis for up-regulated (**a**) and down-regulated (**b**) genes in the *Chilense*_Treated_vs_DVRT-1_Treated comparison group for Biological Process. (**c,d**) The enriched GO terms from the REVIGO analysis for up-regulated (**c**) and down-regulated (**d**) genes in the *Chilense*_Treated_vs_DVRT-1_Treated comparison group for Molecular Function. Circles in closer proximity have more closely related GO terms. The size of the circles indicates the number of child GO terms. The color of the circle represents the significance of the enriched GO terms.

REVIGO tools were used for summarizing the functional categorization of biological process (BP) and molecular function (MF) of top 15 GO terms enriched in DEGs of the *S. chilense*_Treated versus DVRT-1_Treated group according to the lowest *p* values^62^. For up-regulated genes in terms of biological process (BP), oxidation–reduction process, response to stress and protein metabolism were the considerably enriched GO terms (*p* < 0.05) (Fig. 2a). Similarly, other previous transcriptome analyses that are onsistent with our results have suggested that the above processes are associated with salt tolerance in several plants^49,52,54^. For down-regulated DEGs in terms of BP, enriched significant GO terms (*p* < 0.05) comprised mainly lipid biosynthesis process, transcription (DNA-template) and DNA repair (Fig. 2b). For the MF GO term with up-regulated genes, transferase activity and protein kinase activity was significantly enriched (Fig. 2c). The enriched augmented significant GO terms for down-regulated DEGs were contained within a catalytic activity and phosphoprotein phosphatase (Fig. 2d). For up-regulated DEGs, 93 GO BP terms and 47 GO MF terms were significantly enriched in *Chilense*_Treated versus DVRT-1_Treated group (Table S13). The outcome of our result suggest the possible mechanism for salinity tolerance through the up-regulation of numerous genes linked with many BP’s and MF’s in *S. chilense* under salinity.

### Identification of key salt tolerance-related genes

**Figure 3:**
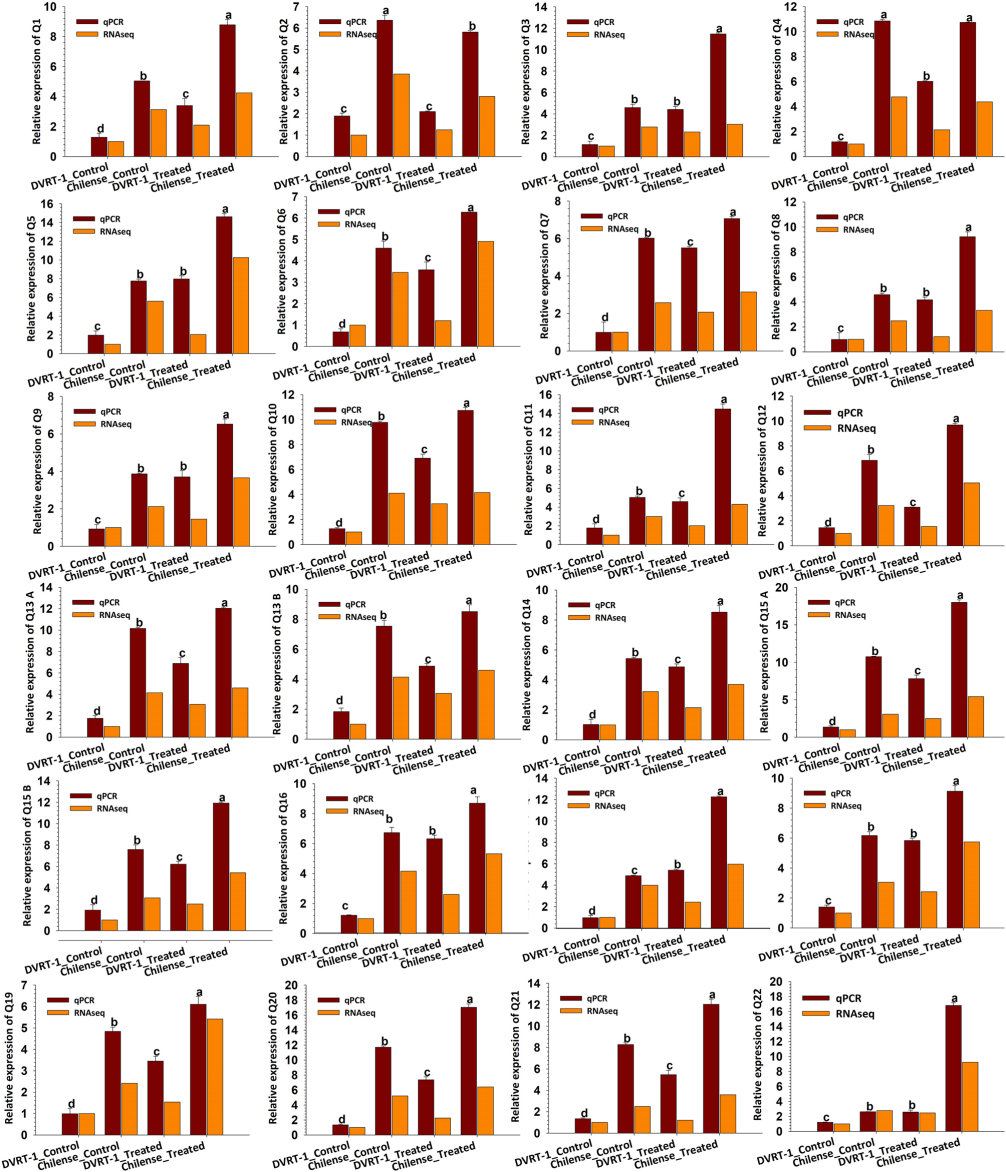
Expression patterns of selected candidate genes related with amine, polyamine metabolism, oxidation–reduction, hormone, ROS scavenging and signaling transduction in *Chilense* and DVRT-1 with and without salt treatment determined by RNA-seq and qPCR. The RNA-seq values represent the ratio of the expression level in *chilense* to the expression level in DVRT-1. Bars with distinct letters are significantly different at *p* ≤ 0.05 applying the DMRT test.

In the present study, numerous salt-responsive genes were identified that could positively contribute to salt tolerance in *S. chilense*. It could be predicted that responsive genes for salt-tolerance displaying differential pattern of expression in the different comparison groups might be playing a key role in salinity tolerance. Subsequently, the overlay among DEGs from the different comparison groups were studied in detail, particularly for the genes which are up-regulated (Tables 1, 2, 3, 4, 5). Validation and confirmation of RNA-seq data were done by qPCR of selected DEGs and gene numbers (Q1–Q69) correspond to the gene labels in Tables 1, 2, 3, 4 and 5 (Fig. 3, Fig. S12 to Fig. S13). Most of the up-regulated genes and major DEGs actively participated in proline and arginine metabolism (Table 1, Fig. 3, Q1 to Q4), oxidoreductase activity (Table 1, Fig. 3, Q5 to Q7), hormone metabolism (Table 1, Fig. 3, Q8 to Q14), ROSscavenging system (Table 2, Fig. 3, Q15 to Q21), signaling regulation (Table 2, Fig. 3, Q22 and Fig. S12, Q23 to Q34), transporters (Table 3, Fig. S12, Q35 to Q43 and Fig. S13, Q44 to Q48), osmotic regulation (Table 4, Fig. S13, Q49 to Q52), defense and stress response (Table 4, Fig. S13, Q53 to Q56), homeostasis (Table 4, Fig. S13, Q57), and transcription factors (Table 5, Fig. S13, Q58 to Q69). Furthermore, genes involved in secondary metabolite biosynthetic process, regulation of systemic acquired resistance, glutathione biosynthesis, glycan metabolism, starch biosynthesis, carbohydrate metabolism, glutathione metabolism, and flavonoid metabolism were also significantly up-regulated (Tables 1, 2, 3, 4 and 5). In our results, we reported down-regulation of polyubiqutin gene in *S. chilense* as well as cultivated DVRT-1. However, this down regulation was more pronounced in *S. chilense* compared to DVRT-1. In one of the studies, Krishnamurthy et al*.*^63^ reported down regulation of polyubiqutin gene during salt stress in halophyte *Avicennia officinalis*. Likewise, we found significant down-regulation of gene encoding pentatricopeptide repeat-containing protein in *S. chilense* compared to its counterparts DVRT-1. In this context, Wang et al.^64^ demonstrated that gene encoding pentatricopeptide repeat-containing protein was down-regulated to provide defense against salinity stress in *Eucommia ulmoides*.

Most of the halophytes have been reported to accrue the greater amount of solutes like glycine betaine, proline, sugars, and polyols against osmotic stress during the salinity stress^65^. In support of this, we detected that the expression of those gene groups which affect the synthesis and metabolites of salt-responsive solutes were significantly higher, for example, glycine betaine biosynthesis encoded by genes *betaine aldehyde dehydrogenase* (*BADH)* and *choline monooxygenase* (*CMO*)^66^, and trehalose biosynthesis encoded by genes *trehalose 6-phosphate phosphatase* (*TPPA*) and *Hexokinase1* (*HXK1*)^67^ in mangrove *(Avicennia officinalis*) treated with salt. Greater accumulation of *CMO* and *BADH* has been reported earlier in many other species such as sugar beet, *Atriplex*, *Suaeda*, *Halogeton*, etc.^68–71^. In the present study too, the expression of *BADH, CMO*, *trehalose 6-phosphate phosphatase* (*TPPA*), and Pyrroline-5-carboxylate reductase were up-regulated in *S. Chilense* (Table 1).

Among genes related with the metabolism of proline and arginine, transcript levels of polyamine oxidase (PAO) genes that are directly involved in proline metabolism were augmented in *S. Chilense* (Table 1). The *q*PCR analysis revealed that the expression patterns of these genes were similar to the transcript levels of the transcriptome data (Fig. 3, Q1–Q4). The transcript levels of the arginine decarboxylase (ADC) encoding the genes regulating the synthesis of putrescine were also identified to have a higher expression in *S. Chilense* (Table 1). Polyamine oxidase and arginine decarboxylase genes play important roles in polyamine and arginine metabolism, and are essential during different environmental stresses^72–75^. Polyamine oxidase has been well-documented in grapevine for drought tolerance improvement^76^. Putrescine plays an important role to minimize the harmful effect of salinity in soybean roots by reducing the oxidative damage^34^. Additionally, numerous studies have depicted that the polyamine catabolism and synthesis are important factors for mitigating the adverse-effect of salinity^77,78^. In our findings, the transcripts expression level of significant genes in polyamine metabolism were augmented in *S. Chilense*, indicating that the polyamine modulation is essential for salt tolerance in S. *chilense*.

Oxidation–reduction processes are another of important factors crucial for salinity tolerance in many plants. GO enrichment analysis revealed that many up-regulated genes were involved in the oxidation–reduction process, which contained the genes mainly associated with glutaredoxin, cytochrome P450 (CYP) and ascorbate oxidoreductase, with relatively higher expression in *S. chilense* (Table 1). Specifically, seven genes that participate in oxidoreductase activity through encoding cytochrome P450 were up-regulated in the *S. chilense* (Table 1). Expression patterns of these genes in *q*PCR analysis were similar to RNASeq transcript data (Fig. 3, Q5–Q7). Cytochrome P450 regulates the activation of molecular oxygen that catalyzes the reaction of bio-oxidation of numerous substrates and controls the metabolic processes of the plant in responses to stress^79^. Higher expression of *CYP94* genes improves jasmonate response which is reported to increase salt tolerance in rice^80^. L-ascorbate oxidase genes which participate in the recycling of ascorbate had higher expression recoded in *S. Chilense* (Table 1). Ascorbate is an essential component for detoxification of cellular H_2_O_2_ through catalytic *Halliwell-Asada* cycle that regulates the reduction of H_2_O_2_ to water and thus play critical roles in plant survival against different stresses^81^. Overexpression of glutaredoxin in tomato plants against salt, drought, and oxidative stresses improves tolerance to abiotic stress^82^. In terms of the entire stress-tolerances scenario, it was concluded that the oxidoreductase process actively participates in ROS scavenging and is important for sustaining homeostasis of oxidation–reduction^83^, and therefore protects the plants from adverse effects of salinity.

### Mechanism of salt tolerance in *S. chilense*

We reasoned that phytohormones, Ca^2+^, ROS, and specific TFs may play critical role in the regulation of different signaling pathways under salt stress. Therefore, total DEGs identified in our study which are expected to be associated with or directly involved in cytokinin, ethylene, auxin ABA and gibberellin signaling pathways were critically analyzed for better understanding of important signaling modules in higher salt stress environments.

#### Role of phytohormone signaling

Hormones like cytokinins, ethylene, jasmonate, and abscisic acid also play a important role in the salinity tolerance regulation mechanism in plants^84^. In term of hormone-related signaling, genes corresponding to jasmonate, ethylene, abscisic acid, auxin, gibberellin, and cytokinins were found to have greater expression in salt-treated *S. chilense,* which may contribute to improved salt-tolerance (Table 1, Fig. 3, Q8 to Q14). Improved salinity tolerance through higher expression of jasmonate content by allene oxide cyclase gene has been reported in wheat and *Arabidopsis*^85^. In consequence of these results, allene oxide synthase 2-like, three lipoxygenase family genes, and one 12-oxophytodienoate reductase gene were found to have augmented expressions displayed in salt-treated *S. chilense*, which are the key genes responsible for the jasmonic acid biosynthesis (Table 1). Similar results are also found in our qPCR analysis that 12-oxophytodienoate reductase, lipoxygenase family genes and allene oxide synthase 2-like gene had considerably greater expressions in the salt-treated *S. chilense* compared to salt-treated DVRT-1 (Fig. 3, Q8, Q13A, Q13B and Q14). Possibly, the augmented expression levels of jasmonate biosynthetic genes under high salinity may play as one of the important factots for enhanced salinity tolerance in *S. chilense*. Makhlouf et al. described the critical role of ethylene response factor genes which are probably related with salinity-tolerance in wheat^86^. Similarly, ethylene response factor genes have also been reported to play a crucial role in response to high salt concentration in different plants^84,87,88^. Regulation and biosynthesis of ethylene by 1-aminocyclopropane-1-carboxylate oxidase gene were up-regulated in the *Chilense*_Treated group (Table 1), which was also confirmed by *q*PCR expression patterns (Fig. 3, Q9). Additionally, the expression pattern of numerous genes related with ethylene biosynthesis like *serine hydroxy methyltransferase* (*SHMT)*, *S-Adenosyl methionine synthetase* (*SAM2*), and *methionine synthase* were detected to be greater in the *Chilense*_Treated group in both the *q*RT-PCR and RNASeq results (Table 2, Fig. S12, Q32–Q34). Moreover, ethylene signaling encoded by *hexokinase*^89^ and proline synthesis encoded by *glutamate synthase*^90,91^ are well-identified genes for salt tolerance (Table 2). Alterations in the expression pattern of ethylene-associated genes suggested that ethylene may act as an important factor for tolerance to salinity in *S. chilense*. Hence, we theorize that the ethylene signaling plays an important role for salt tolerance in *S. chilense* under high salinity. In the present study, many genes associated with auxin biosynthesis and metabolism such as *14-3-3 protein zeta* were up-regulated (Table 2, Fig. S12, Q28). This strongly indicates that the auxin signaling was highly involved in salinity tolerance in *S. chilense*. Besides, genes involved in biosynthesis or regulation of abscisic acid, cytokinin, auxin, and gibberellin showed higher expression levels in salt treated *S. chilense.*

#### Role of ROS

Several genes that regulate the activity of enzymes and are involved in detoxification and scavenging of reactive oxygen species (ROS) during salt stress were found up-regulated in *S. chilense*, in support of earlier reports^92,93^ (Table 2 Fig. 3, Q15 to Q21). These contain superoxide dismutase, catalase, peroxidases, glutathione peroxidase, glutaredoxin, and thioredoxin reductase. Up-regulation of antioxidant genes revealed that the severe oxidative stress is prompted by salt stress, and thus scavenging and ROS detoxifying enzymes are responsible for tolerance and metabolic adaptation with respect to salinity in *S. chilense*. Significant up-regulation of antioxidants is also noticed in various crops under salt stress like jatropha, calendula, tomato, pea, maize seedlings, and bean seedlings^94–102^.

#### *Role of Ca*^*2*+^*signaling*

Various types of signal transduction genes were up-regulated in *S. chilense* (Table 2). These transcript results were validated by the *q*PCR analysis of selected genes (Fig. 3, Q22 and Fig. S12, Q23–Q34). Genes encoding signal transduction function such as CBL-interacting protein kinases (CIPK), protein histidine kinase, protein serine/threonine kinase, calmodulin, calcium/calmodulin-dependent protein kinase along with other genes related to signal transduction were up-regulated in *S. chilense* (Table 2). In *Arabidopsis*, the network cascade of CBL/CIPK has been well-known for regulation of Na^+^ efflux transporter SOS1 activity^17,103,104^. Numerous genes associated with CIPK in many plants have already been established for their salt tolerance function^18,104,105^, and it is assumed that the higher expression level of CIPK genes endorses greater tolerance under salinity environment in *S. chilense*.

Under different abiotic stresses, the most vital second messenger for plant signaling networks is calcium. Various stress-stimuli (such as salt stress) results in an upsurge of Ca^2+^ levels in the plant cytosol within few seconds by means of Ca^2+^ pumps and transporters^63^. In our study, many genes associated with the signaling of Ca^2+^ were up-regulated (Tables 2, 3). Ca^2**+**^/H^+^ exchangers (*CAX*s), cyclic nucleotide-gated channels (*CNGC*s), and calcium-transporting ATPases (*ACA*s) were significantly up-regulated in *S. Chilense*, which could be the main factor of the Ca^2+^ fluxes during salinity (Table 3, Fig. S12, Q39 and Q43). Cyclic nucleotide-gated channels (*CNGC*s) are accountable for the uptake of Ca^2+^, Na^+^, and K^+^, whereas Sodium/hydrogen exchanger or NHXs are known to be involved in the compartmentalization of K^+^, Na^+^, and pH homeostasis. All these functions are regulated by pH gradient produced by V-ATPases^106^. K^+^ transporters also play an important role in ionic balance process which is otherwise hampered due to salinity stress^107^. ABC transporters are concerned in several functions comprising transport of Na^+^, osmolytes, heavy metals, auxin and fatty acids^108–110^. While the major role of cyclic nucleotide-gated channels during salt stresses is to influx the Ca^2+^ ion across the plasma membrane, the Ca^2+^/H^+^ exchangers and calcium-transporting ATPases perform efflux process of Ca^2+^ across the plasma membrane and tonoplast, correspondingly. As a result of the higher level of Na^+^/Cl^-^, the concentration level of Ca^2+^ ion is increased and sensed immediately by the calcium sensors like CBLs, CIPKs, CAM kinases, and SOS3^103,111^, all of these being significantly up-regulated in *S. Chilense* (Table 2, Fig. S12, Q26, Q27, Q29A, Q29B and Q31). Further, Ser/Thr protein kinases (CIPKs/SOS2) interact with SOS3 resulting in formation of a specific SOS3-SOS2 complex that is adequate to trigger several downstream targets against salinity^112^. The NHX1 and Na^+^/H^+^ antiporters SOS1, triggered by the activated SOS3–SOS2 complex initiates efflux of Na^+^ across the plasma membrane and compartmentalization of Na^+^ into vacuoles at the same time^112,113^. Additionally, HKT is known to prevent the entry of Na^+^ in the roots, thus providing salt tolerance in plants. In our study, it was found that both HKT and Na^+^/H^+^ antiporter activities were up-regulated in *S. Chilense* (Table 3, Fig. S13, Q48); whereas many other regulatory components were also highly up-regulated in *S. Chilense* (Table 3, Fig. S12, Q35 to Q43 and Fig. S13, Q44 to Q48). Besides these, calcium ion binding proteins encoded by *calmodulin* genes and Ca^+^/H^+^ exchangers also proved their significant roles in salt tolerance in *S. chilense* (Tables 2, 3). Comprehensively, our results indicate that signaling of Ca^2+^ play a crucial role in salt tolerance in *S. chilense* in addition to the role of salt-responsive phytohormones.

#### Role of transcription factors

Most of the biological processes, specifically stress response is under direct influence by transcriptional gene expression^114^. In the current study, a total of 6,353 TFs were identified that grouped into 57 TFs family (Table S7). In our study, more than twelve different types of TFs and salt-induced genes encoded by their-respective proteins were recorded in higher expression in salt-treated *S. chilense* compared to the salt-treated DVRT-1 plants (Table 5), advising that these TFs may contribute to more tolerance against salinity conditions in *S. chilense*. The TFs *bZIP*, *MYBs*, *bHLH*, *C2H2-Dof*, *G2-like*, and *AP2-EREBP* are well-known to play a major role in salt-stress responses of common bean, *Oryza sativa, Raphanus sativus*, *A. thaliana,* and *Populus trichocarpa*^22–24,115^. In our findings, TFs from the *Dof* zinc finger protein, *C3HC4*-type zinc RING finger, *WRKY*, *bHLH, TCP* transcription factor 23, *NAC*, *GATA*, *ARF*, *ERF*, *bZIP*, and *MYB* families were significantly up-regulated in *S. chilense*, proposing that these TFs must be regulating salt-stress response in *S. chilense* (Table 5, Fig. S13, Q58 to Q69).

Role of *bHLH* TF in salinity response has been reported earlier in other plant species^116^. Up-regulation of *bHLHs* TFs with diverse peroxidase antioxidants genes in the present study proposes their possible participation in peroxidase-mediated ROS detoxifying-regulation under salt treatment in *S. chilense*. Involvement of *bHLH* TFs in SOS1 regulation through genes encoding Na^+^/H^+^ plasma membrane antiporter is important for salt tolerance and has been documented in other plants^117^. Shi and Chan ^118^ reported that the expression of *AtZAT6* and *C2H2* zinc-finger protein can be triggered by salt, drought, and cold stresses in *Arabidopsis*. In compliance with this, a total of 11 different zinc-finger TFs were up-regulated in our experiments suggesting the possible role of zinc-finger proteins in salt tolerance in *S. chilense*. *MYB* TFs are involved in secondary metabolism, abiotic stress tolerance, hormone signal transduction, and disease resistance in plants^119^. *MYB59*, *MYB48-like*, *MYBR1*, *MYB76*, and *MYB12* were found to have higher expression under salt stress (Table 5), and could play a crucial role in the salt tolerance in *S. chilense*. Besides these, *WRKY* TFs also actively participate in the regulation of abiotic stress process and have been reported in several crops. In tomato, *SlWRKY3* is significantly up-regulated by the induction of drought and salt stress^120^. In soybean, 25 *WRKY* genes are involved in drought and high salt stress treatment^121^. Similar to these results, three *WRKY* TFs , viz., *WRKY1*, *WRKY3* and *WRKY72* displayed observably higher expression during salt stress in our study (Table 5), justifying the significant role of *WRKY* TFs in salt tolerance of *S. chilense*. Earlier studies have successfully established the correlation among the DEGs from transcriptome and *q*PCR data^49,122^. The validation of selected genes through *q*PCR revealed the similar expression patterns corresponding to the DEGs from transcriptome data, but the fold changes were not accurately the same.

Overall, the findings of our experiments directly advocate that different genes, which are actively-involved in ion homeostasis, phytohormone, oxidation–reduction, ROS scavenging, detoxification, signaling transduction, transporters, osmotic regulation, defense and stress response, and other metabolic processes are significantly up-regulated, and play important role in salt tolerance of *S. chilense*. The functionality of salt-responsive genes identified in the present study is required to be ascertained for their further validation through different experimentation. With the support of comparative transcriptomic approach, strategic salt tolerance-responsive genes in *S. chilense* were identified, and the chronological expression patterns of selected genes were validated through *q*RT-PCR. In addition to Ca^2+^ signaling, phytohormones and transporters are well known to portray crucial roles in plants salt tolerance^123^. The vacuolar ATPases and NHXs also play important roles in the homeostasis of Na^+^/K^+^ in *S. chilense*, with their functions much similar in other plants^124^. In the same way, up-regulation of numerous genes related to signaling pathways emphasized their significance and importance in *S. chilense.*

## Conclusion

In this study, a comparative transcriptomic analysis of *S. chilense* and cultivated tomato, in response to 500 mM NaCl was carried out to identify the various genes and pathways associated with salinity stress, as well as to deliver a valuable catalog of RNA-Seq difference caused by salinity. Our results have revealed the interactions among the genes involved in Ca^2+^, auxin, and ethylene mediated signaling network in response to salt-stress. This information will provide worthy genomics and molecular knowledge for future research on salt tolerance in *S. chilense* and other related species of tomato.

## Materials and methods

### Plant materials, growth conditions, and salt treatment

Two tomato species, viz., cultivated tomato (*S*. *lycopersicum* L.) cv. DVRT-1/Kashi Amrit, and a wild relative of tomato (*S. chilense* L.) were used in this experimental study. Seeds of *S. chilense* (LA1972) were provided by Cranfield University, MK 43 0AL, United Kingdom. Seeds of both tomato genotypes were sown and raised inside a greenhouse (25.3521° N, 82.9502° E) at ICAR-Indian Institute of Vegetable Research, Varanasi, India. Tomato is a grown as a transplanted crop and 25–30 days old seed lings are transplanted in the main field. Tomato plants are more likely to be exposed to salinity in the main field than in nursery. Therfore, 1-month-old seedlings were transplanted into small pots (one pot/plant) filled with soil, mixture of vermiculite-perlite (1:3), and farmyard manure (5:1) under greenhouse conditions: 26 °C, 16 h for day, and 15 °C, 8 h for night, and 55% to 65% relative humidity. In order to reduce plasmolysis caused by the osmotic shock, NaCl was gradually increased at a rate of 50 mM each day until the desired concentration (500 mM) was reached (Fig. S14). A control treatment (0) was carried out without NaCl solution with electrical conductivity (EC) 3.8 dSm^–1^. NaCl treated seedlings were carried out with 500 mM NaCl solution with EC 26.8 dSm^–1^. 21 days after reaching 500 mM NaCl, samples were collected for RNA sequencing from the NaCl treated and control plants viz. *S. chilense* and *S. lycopersicum* plants. For each treatment, 9 seedlings (three plants from three replicates) each from the control and treated were harvested after 21 days of salt treatment.

### RNA extraction and cDNA library preparation

Tissue samples from three plants for each treatment were pooled and ground to fine powder to create one pooled sample. One sample from each treatment was used for Illumina sequencing. Further, two technical replicates for each treatment derived from pool sample were used for total RNA isolation and qRT-PCR analysis. 200 mg ground leaf tissue was used to isolate total RNA using RNeasy Plant Mini Kit (Qiagen, USA) as per the protocol suggested by the manufacturer. RNA purification kit and DNase I (NEB, USA) were used for removing DNA contaminations from the total RNA. The purity and quality of RNA samples were measured on agarose gel electrophoresis and in a Nano-spectrophotometer (Implen, USA). Integrity of RNA was checked using the Nano 6000 Assay Kit with the Agilent 2100 Bioanalyzer (Agilent Technologies, USA). RNA concentration was evaluated using a Qubit RNA Assay Kit with Qubit 2.0 Fluorometer (Life Technologies, USA). RNA Library Prep Kit NEBNext Ultra for Illumina (NEB, USA) was used for the construction of sequencing libraries following the recommendations of the manufacturer. Further, Oligo(dT)-attached magnetic beads were used for mRNA purification. The cDNA library was prepared after fragmentation, and hybridization process was completed with ligation of NEBNext hairpin loop adaptors (Illumina, USA). AMPure XP system (Beckman Coulter Life Science, USA) was used in PCR products purification, and cDNA library quality was checked on an Agilent 2100 Bioanalyzer. Finally, a total of four cDNA libraries [For each individual treatments (salt treated and control)] were successfully assembled.

### Sequencing and transcriptome de novo assembly

To identify and gain knowledge on the genetic and molecular mechanisms of salt tolerance in tomato species, four cDNA libraries were constructed. It included one assembly for each individual treatments of *S. chilense* accession and cultivated tomato cultivar (*Chilense*_Control, *Chilense*_Treated, DVRT-1_Control, and DVRT-1_Treated). Thus obtained cDNA libraries were deep sequenced using Illumina HiSeq 2000 sequencer to produce paired-end reads (150 bp). Illumina data processing pipeline^125^ was used to check the quality of raw reads. The rawreads were pre-processed to get the high-quality clean reads. The raw reads were filtered by removing reads with adaptor sequences, reads containing duplicated sequences, reads covering more than 10% ambiguous base reads (“N”), and low quality reads with more than 50% of low-quality bases (Q-value ≤ 10). All downstream assembly analyses were done on processed high-quality reads. De novo assembled contigs were generated from the clean reads using a de novo transcript assembly tool Trinity^126^. Redundancy was removed by TGICL software^127^, and thus the sequences were assembled into a non-redundant single contig. The longest contigs of chimeric and redundant transcripts were referred as unigenes (viz. 25 k-mer lengths), and were exposed to the downstream coding sequence (CDS) and functional annotation prediction. Assembled unigene sequences were aligned using following databases: BLASTx (E-value ≤ 1E-5) (https://www.ncbi.nlm.nih.gov/BLAST/) contrary to NCBI database of non-redundant protein (nr) (https://www.ncbi.nlm.nih.gov)^128^, the KEGG database (https://www.genome.jp/kegg)^129,130^, the Viridiplantae, and the KOG/COG database (https://www.ncbi.nlm.nih.gov/COG)^131^. ESTScan^132^ was used to align sequences for those unigenes that could not be aligned to any of the above databases. The RNA-Seq raw data generated from this study was submitted to the Sequence Read Archive (SRA), National Centre for Biotechnology Information (NCBI) website with the accession numbers PRJNA559982 and PRJNA560126.

### Data analysis

To assign the gene functions of identified unigenes, sequences were explored in the aforesaid public databases with BLASTx program. Proteins with highest sequence similarity to the *S. chilense* and *S. lycopersicum* unigenes along with their functional annotations were retrieved. Gene Ontology (GO) annotation was completed after annotation of nr using software Blast2GO^133^. PANTHER14.1 tool was used to carry out functional classification of the unigenes and to assign the GO distribution of gene functions at the broad level^134^. The unigenes were further aligned using KEGG and COG/KOG annotation to get pathway information for an identified orthologous gene. For unigenes mapping, the clean read sequences were mapped deploying Bowtie version 2.1.0^135^ and Tophat version 2.0.8^136^ on tomato genome SL 2.5 (version 2.50) https://solgenomics.net/. Further, quantification of the reads abundance per each gene (exon level) was performed using AIR (https://transcriptomics.sequentiabiotech.com/) and were obtained through iTAG gene annotation (version 2.5). Gene expression analysis was carried out using reads per kilobase per million reads (RPKM) method^137^. Differential expression analysis was performed using the DESeq R package (1.18.0). False discovery rate (FDR) ≤ 0.001, the absolute value of log2Ratio ≥ 2 and *p*-value ≤ 0.001 was used as the threshold to judge the significance of differential gene expression in salt-treated and untreated samples of both *S. chilense* and *S. lycopersicum*^138^. For GO enrichment and pathway analysis, all the DEGs were mapped in GO and KEGG databases (https://www.geneontology.org/) and further analysis was completed with GOseq R package. Enrichment analysis of KEGG pathways of DEGs was done by KOBAS software^139^. DEGs sequences were further aligned to the published microarray/transcriptome data to categorize the important genes related to salt-tolerance in *S. chilense* and *S. lycopersicum*.

### Identification of transcription factor families

The unigenes sequence was analyzed using online tool PlantTFcat (https://plantgrn.noble.org/PlantTFcat)^140^ to identify the putative TFs families in the public domain transcriptome databases.

### SSRs identification

The identification and distribution of various types of simple sequence repeats (SSRs) were analyzed using the MIcroSAtellite tool (MISA version 1.0; https://pgrc.ipkgatersleben.de/misa/misa.html)^141^.

### Quantitative real-time PCR (qRT-PCR) analysis

For analysis of quantitative *q*RT-PCR, the first-strand cDNA was synthesized from one microgram of total RNA with iScript cDNA synthesis kit (Bio-Rad Laboratories, USA) according to manufacturer’s protocol. SsoFast EvaGreen Supermix (BioRad Laboratories, USA) was used to study expression analysis of total 69 salt stress related genes using *q*RT-PCR following the manufacturer's instructions with an iQ5 thermal cycler (BioRad Laboratories, USA). The primers used for *q*RT-PCR are detailed in supplementary Table S14. Quantitative PCR reactions were performed in 8 strips optical flat PCR tube and each reaction mixture containing 10 μl of SsoFast EvaGreen Supermix (2**×**), 1 μl of specific forward and reverse primers (200 nM), 2 μl of the reverse-transcribed cDNA template (25 ng), and 6 μl ddH_2_O. The *q*RT PCR conditions were performed with following steps: 95 °C for 10 min, followed by 45 cycles at 95 °C for 15 s, at 55 °C to 60 °C (primer melting temperature) for the 30 s, at 72 °C for 30 s and finally one cycle at 72 °C for 5 min. Every reaction was repeated three times and the relative level of gene expression quantification was calculated using the 2^−ΔΔCT^ method^131^. NormFinder software was used for analysing the expression stability check values of reference genes, which is based on variance analysis to calculate the stable value of each gene^142^. According to the stability values of reference genes calculated by NormFinder (Table S14), Actin (0.142) was found the most stable genes under salinity stress condition. Constitutively expressed actin gene was used as internal control for normalization of the Ct value.

## Supplementary information

Supplementary Information.

Supplementary Table 3.

Supplementary Table 4.

Supplementary Table 6.

Supplementary Table 7.

Supplementary Table 8.

Supplementary Table 9.

Supplementary Table 10.

Supplementary Table 11.

Supplementary Table 12.

Supplementary Table 13.

Supplementary Table 14.
