# Supplementary material for: Understanding salt tolerance mechanism using transcriptome profiling and de novo assembly of wild tomato Solanum chilense
Source: Sci Rep. 2020 Sep 28;10:15835. doi: 10.1038/s41598-020-72474-w (PMC7523002; doi:10.1038/s41598-020-72474-w)
Supplement: Supplementary file 1 — Supplementary Information. [file 41598_2020_72474_MOESM1_ESM.pdf]

Supplement data in brief

Title:

Understanding salt tolerance mechanism using transcriptome profiling and de novo assembly of wild tomato *Solanum chilense*

Authors:

SP Kashyap[1, 2], HC Prasanna[1, 3*], Nishi Kumari[2], Pallavi Mishra[1] and B Singh[1]

Author's affiliations:

[1] Division of Crop Improvement and Biotechnology, Indian Institute of Vegetable Research, Indian Council of Agricultural Research, Varanasi-221 305, Uttar Pradesh, India

[2] Department of Botany, Mahila Maha Vidyalaya, Banaras Hindu University, Varanasi-221 005, Uttar Pradesh, India

[3] Division of Vegetable Crops, Indian Institute of Horticultural Research, Indian Council of Agricultural Research, Hessaraghatta, Lake Post, Bengaluru-560 089, Karnataka, India.

[*]Corresponding author:

Dr. HC Prasanna, Principal Scientist, Genetics and Plant Breeding,

Division of Crop Improvement and Biotechnology, Indian Institute of Vegetable Research, Indian Council of Agricultural Research, Shahanshahpur, Jakhini, Varanasi-221 305, Uttar Pradesh, India

E-mail: prasanna.c@icar.gov.in, prasanahc@yahoo.com

Mobile: +91-9455178097

E-mail address of authors:

singhsarvesh10@gmail.com (SP Kashyap); prasanna.c@icar.gov.in (HC Prasanna); kumaridrnishi@yahoo.co.in (Nishi Kumari); pallavimishra23@gmail.com (Pallavi Mishra);bsinghiivr@gmail.com (B Singh)

Supplemental Fig. S1

Distribution of unigenes length. Among these unigenes, 35,4571 unigenes were longer than 300 bp, 72,904 unigenes were longer than 500 bp and 27,431 unigenes were longer than 700 bp.

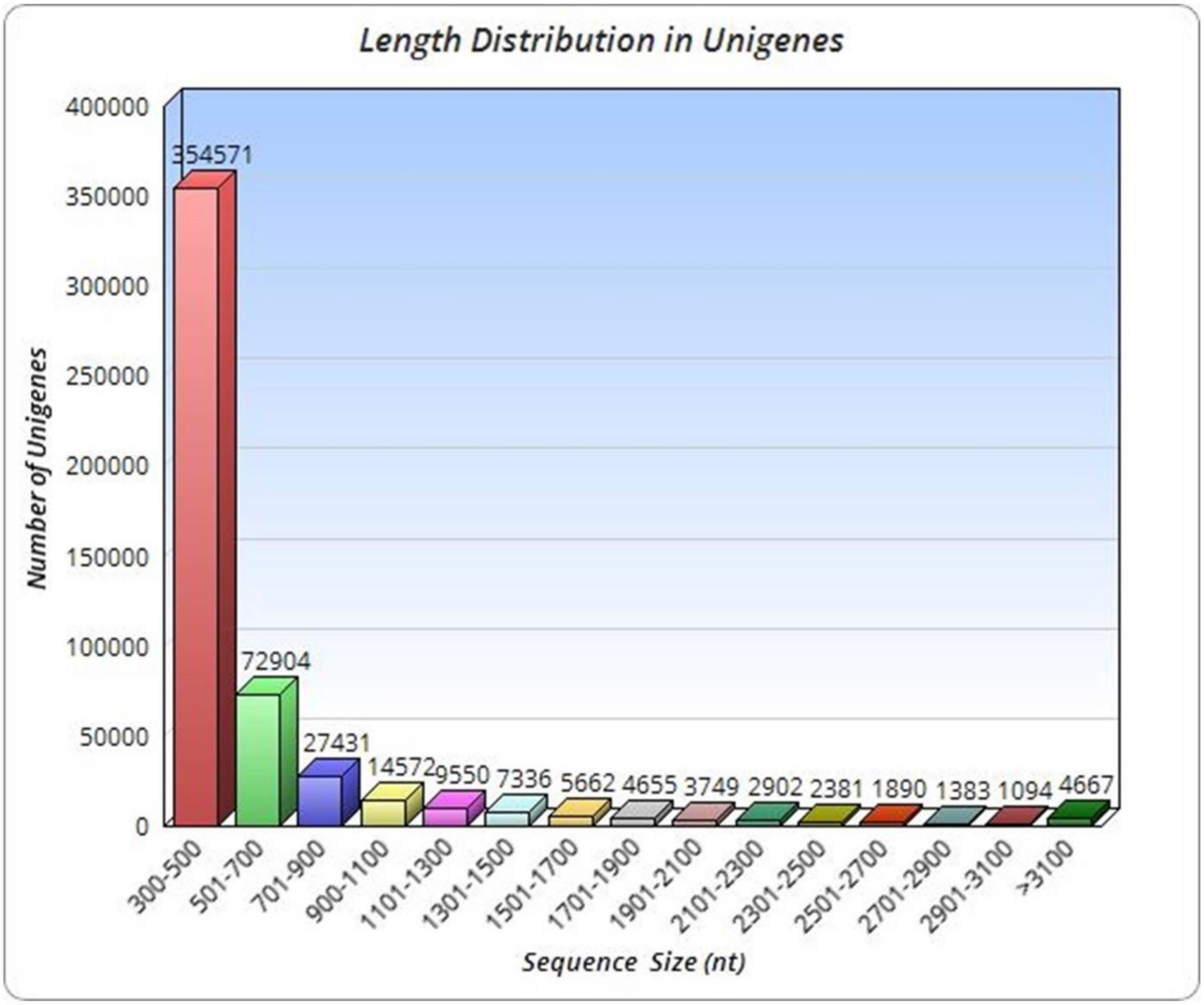

Supplemental Fig. S2a

Distribution of unigenes length (*Chilense*_Control). Among these unigenes, 99,461 unigenes were longer than 300 bp, 22,061 unigenes were longer than 500 bp and 9,921 unigenes were longer than 700 bp.

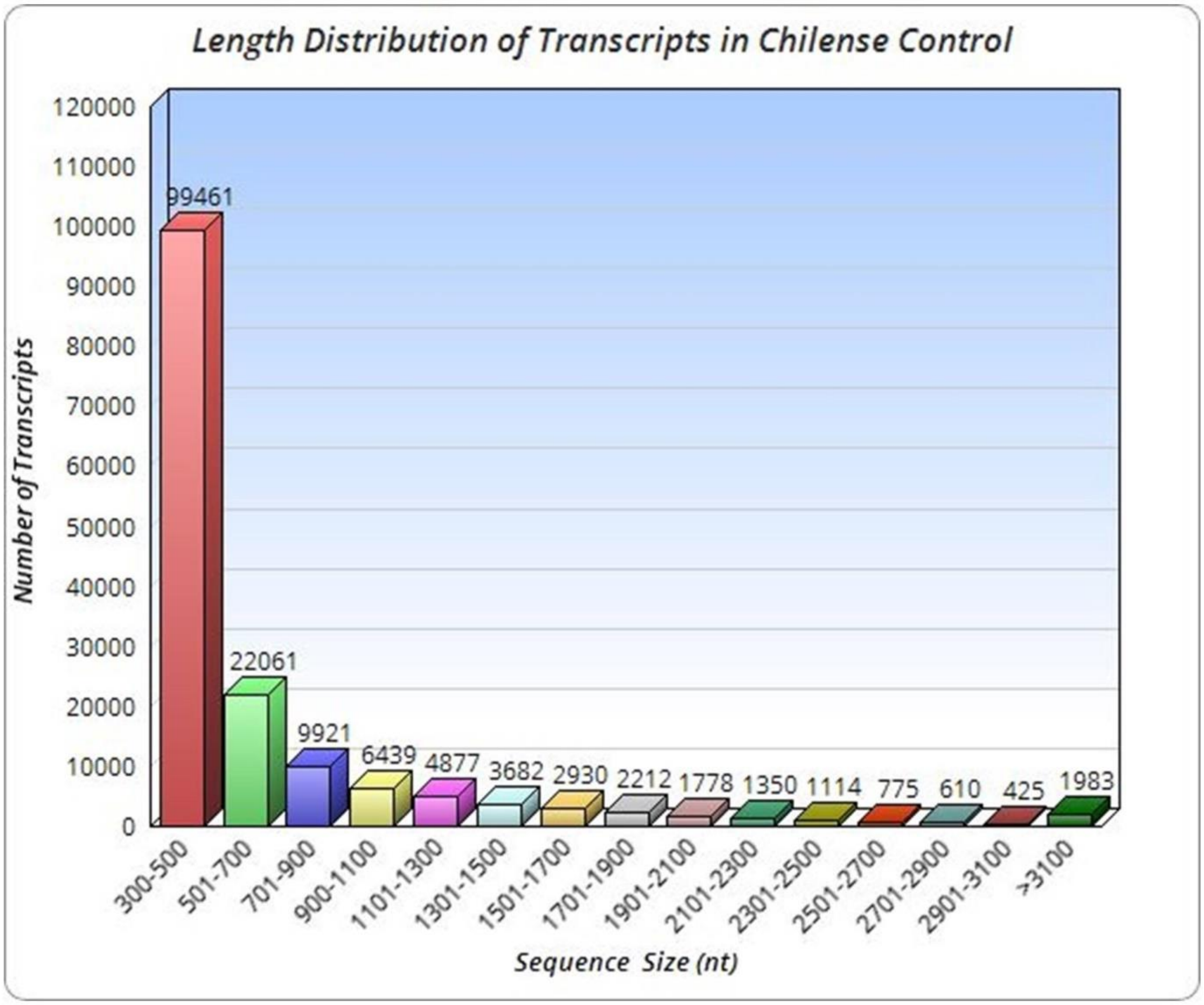

Supplemental Fig. S2b

Distribution of unigenes length (*Chilense*_Treated). Among these unigenes, 1,10,974 unigenes were longer than 300 bp, 24,744 unigenes were longer than 500 bp and 10,755 unigenes were longer than 700 bp.

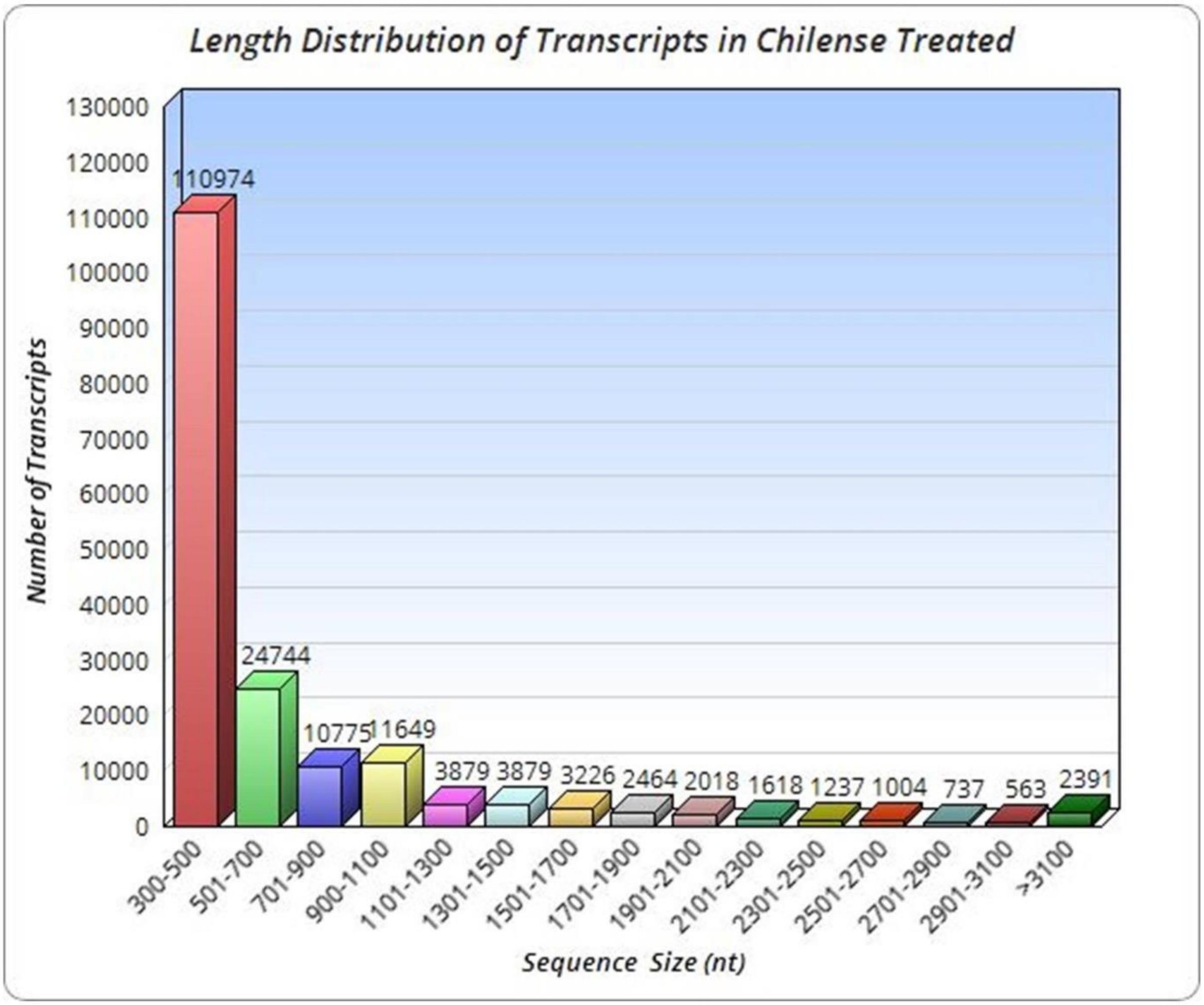

Supplemental Fig. S2c

Distribution of unigenes length (DVRT-1_Control). Among these unigenes, 1,55,345 unigenes were longer than 300 bp, 35,623 unigenes were longer than 500 bp and 14,655 unigenes were longer than 700 bp.

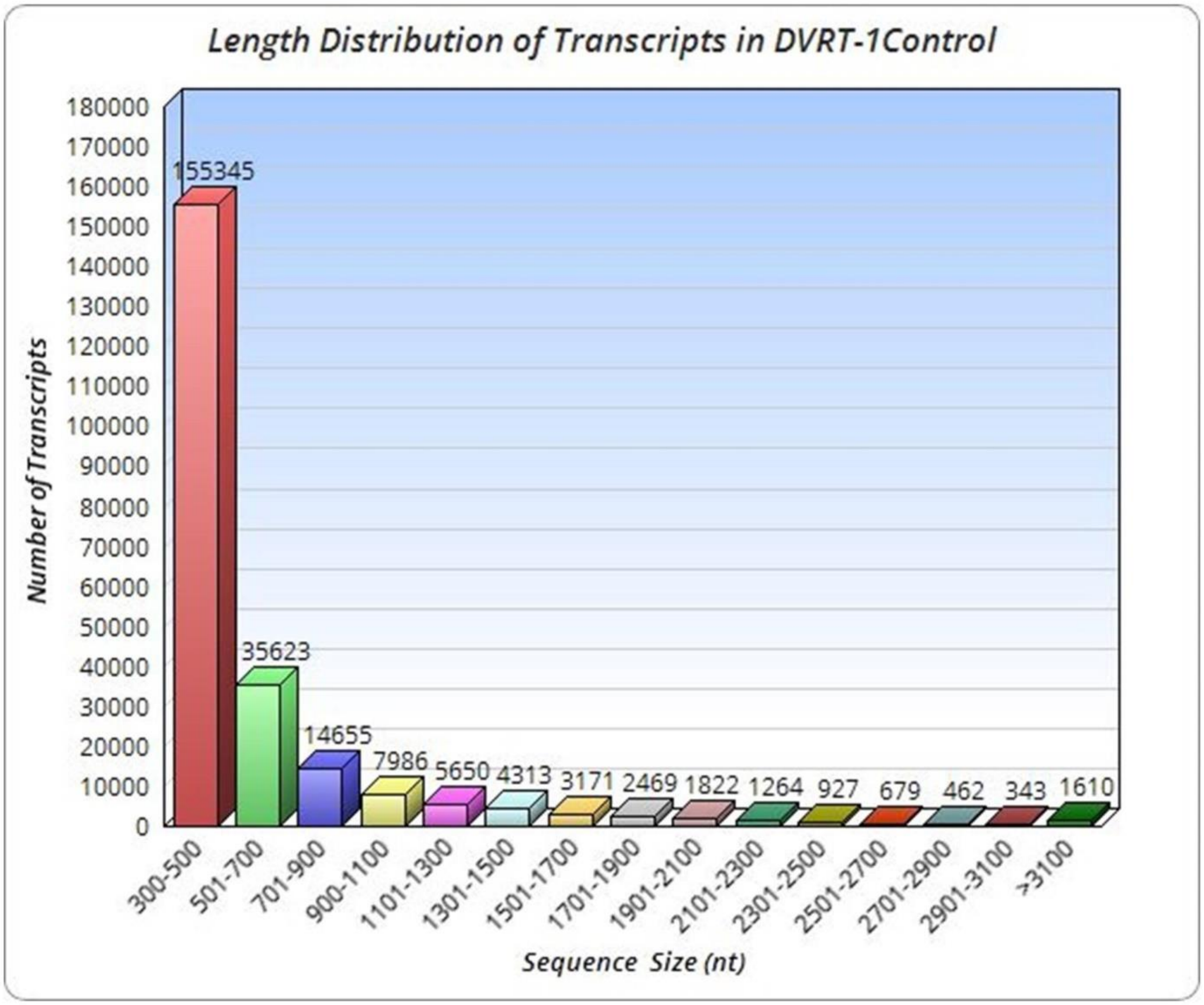

Supplemental Fig. S2d

Distribution of unigenes length (DVRT1_Treated). Among these unigenes, 1,37,447 unigenes were longer than 300 bp, 35,806 unigenes were longer than 500 bp and 17,021 unigenes were longer than 700 bp.

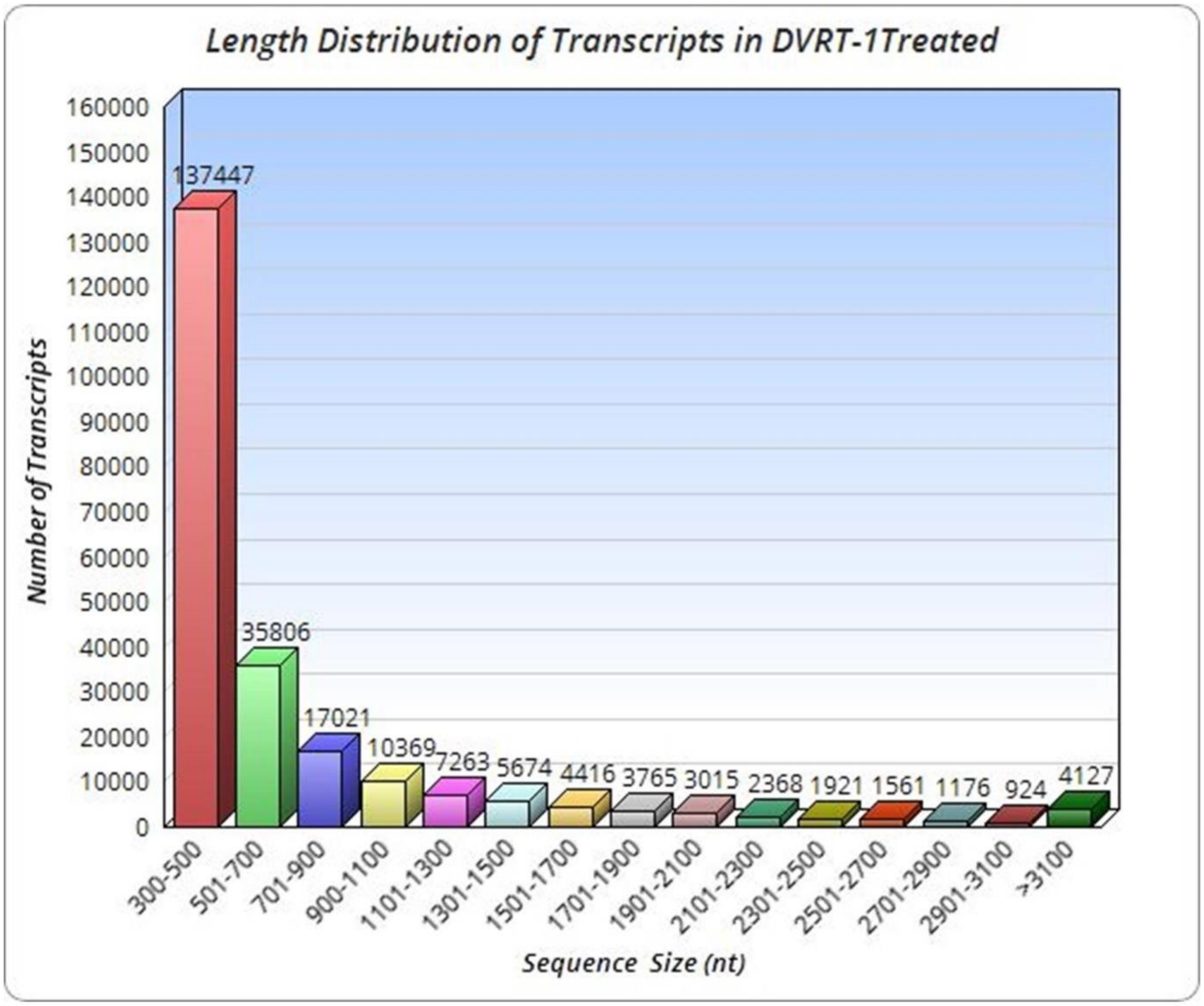

Supplemental Fig. S3

Function annotation of assembled transcripts based on Gene Ontology (GO) analysis. According to their sequence homology, a total of 1,08,355 unigenes were assigned into 3 main GO categories viz., cellular component (CC), biological process (BP) and molecular function (MF).

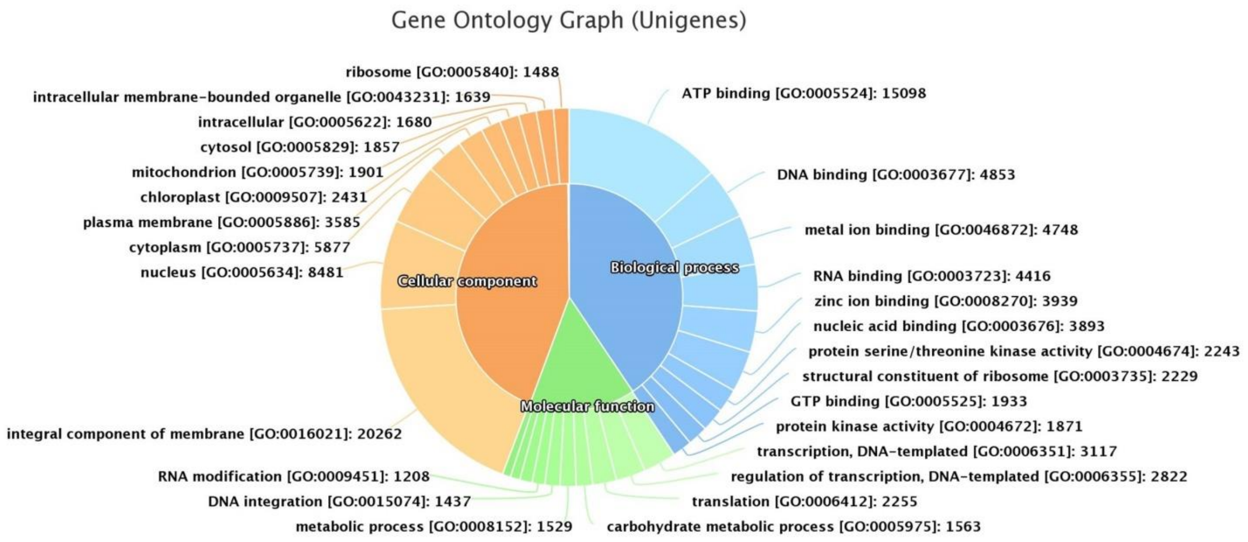

Supplemental Fig. S4

Pathway assignment based on the Kyoto Encyclopedia of Genes and Genomes (KEGG) database. A total of 7,923 unigenes were mapped into the reference canonical pathways and assigned to 127 KEGG pathways, and further divided into five different KEGG functional groups viz., cellular processes, environmental information processing, genetic information processing, metabolism and organismal systems.

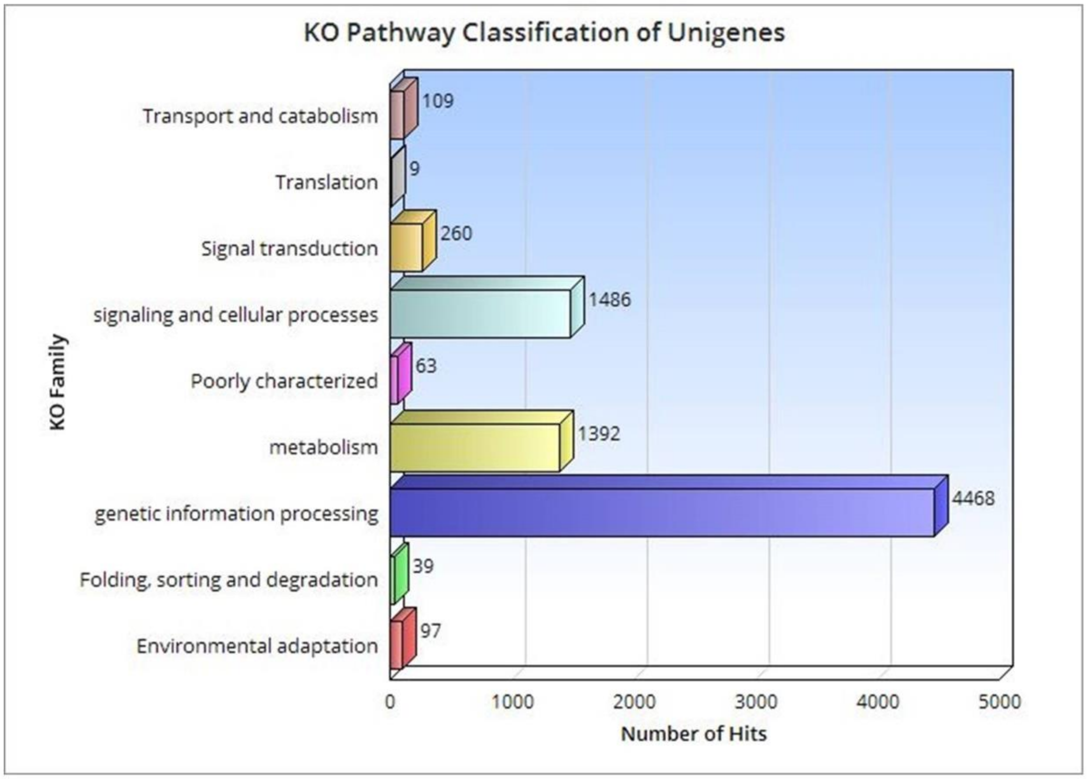

Supplemental Fig. S5

Classification of assembled transcripts based on the euKaryotic Ortholog Groups (KOG) database. A total of 1,26,937 unigenes were assigned into 26 diverse functional categories.

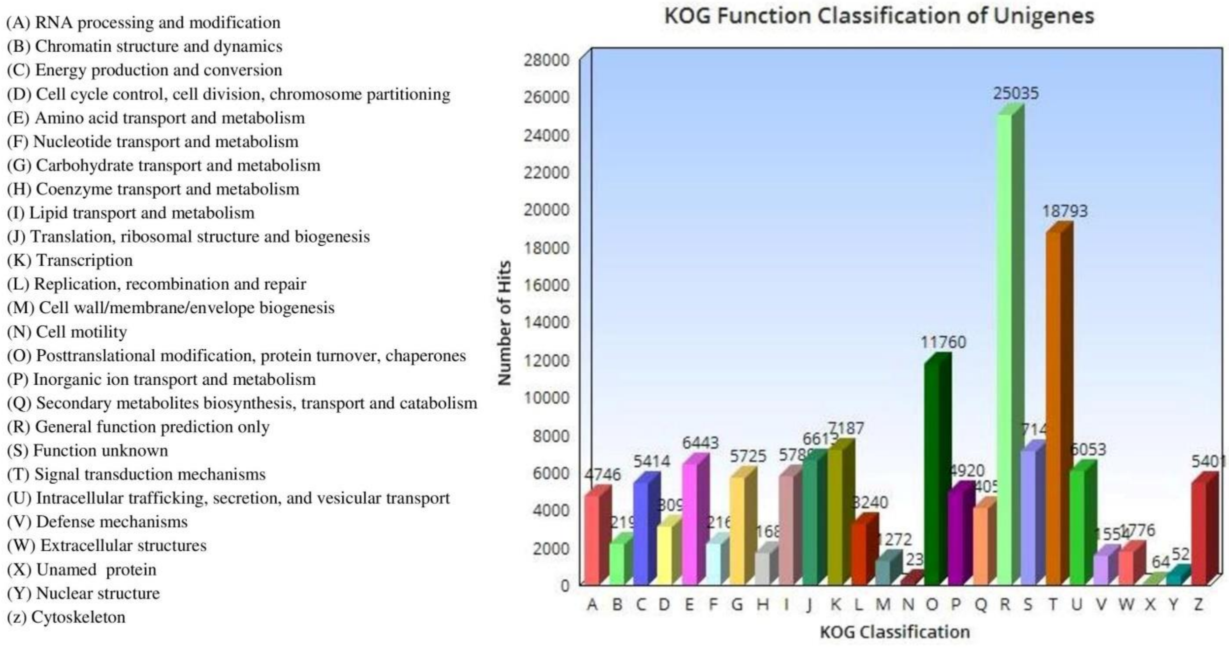

Supplemental Fig. S6

Distribution of SSRs motif based on SSR unit size. A total of 1,06,239 potential SSRs were classified based on the SSR unit size.

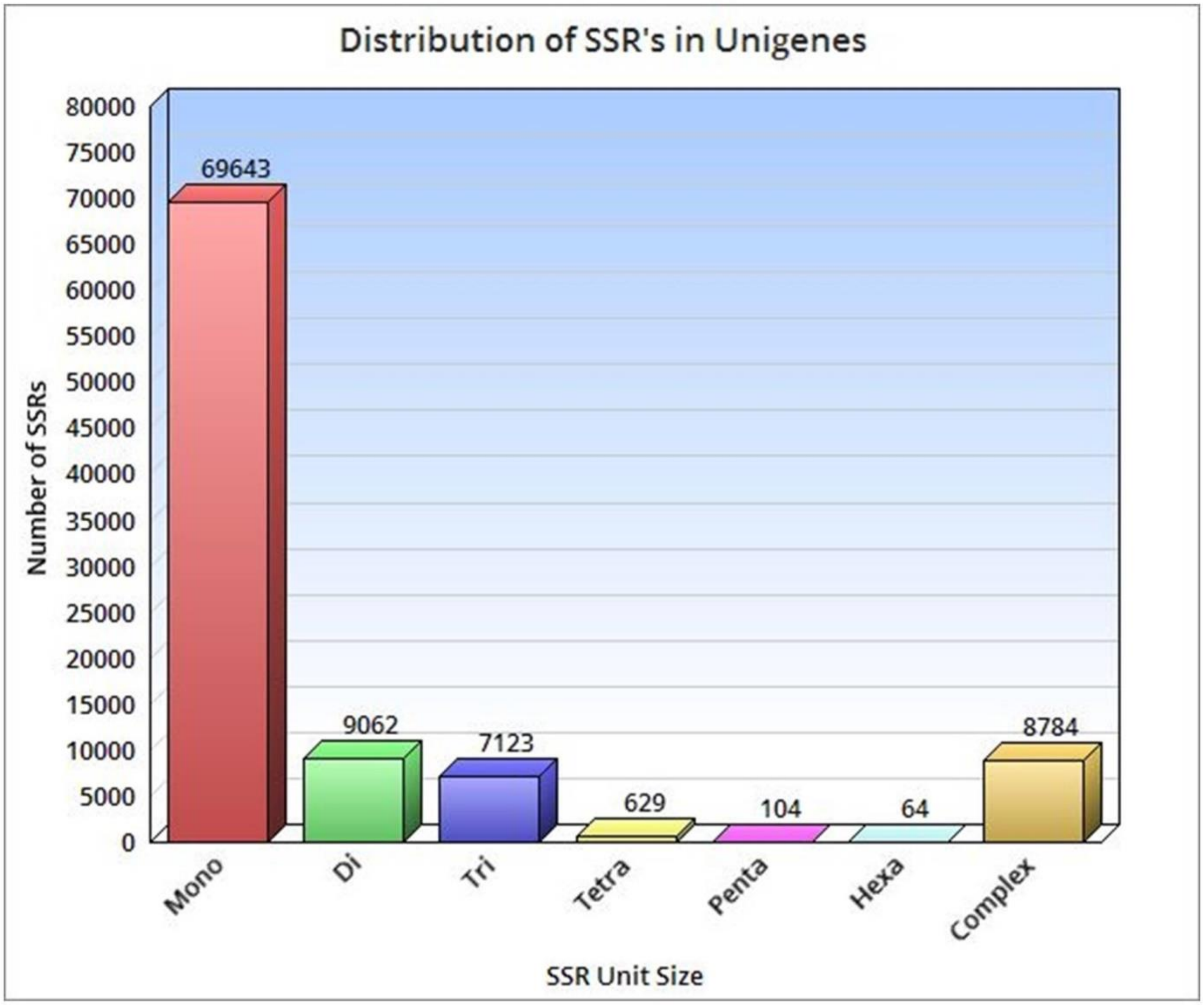

Supplemental Fig. S7

Volcano plot of the differentially expressed genes shows the estimated log2 (fold change) (x-axis) against its statistical significance (y-axis) between control and treated groups. (**a-c**) DEGs between salt treated *chilense* and chilense (*Chilense*_Treated vs Control, **a**), salt treated DVRT 1 and DVRT 1 (DVRT-1_Treated vs Control, **b**), salt treated *chilense* and salt treated DVRT-1 (*Chilense*_Treated vs DVRT-1_Treated, **c**) displayed by volcano plots. The abscissa displays the fold change difference in the expression of genes in different comparison groups, and the vertical coordinates indicate the adjusted P-values for the differences in expression. Genes without significant differences are indicated by black dots. The up-regulated genes are represented by red dots, and the down-regulated genes are represented by green dots.

Fig S5a

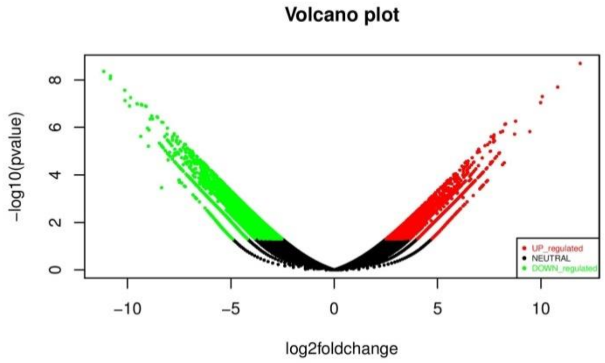

Fig S5b

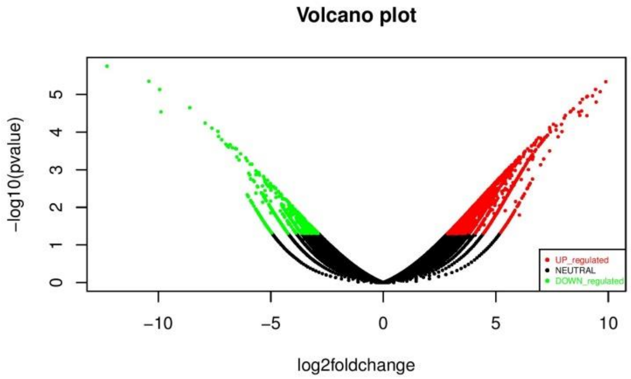

Fig S5c

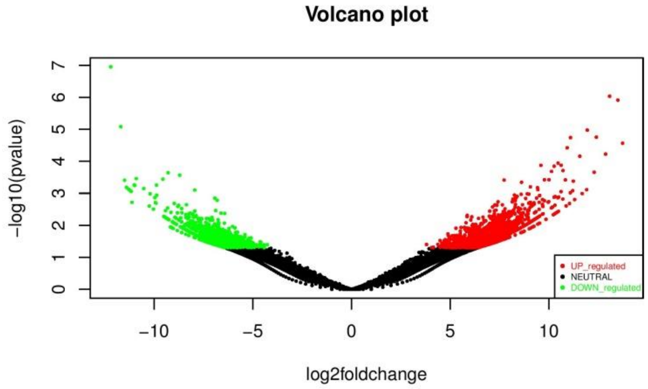

Supplemental Fig. S8

Heatmap and clustering of differentially expressed genes (DEGs) between the salt-treated and untreated groups. Each column represents a different sample. Colour bar indicates the relative expression level from up-regulated (red) to down-regulated (green). Heatmap was created using Heatmapper online tool (http://heatmapper.ca/).

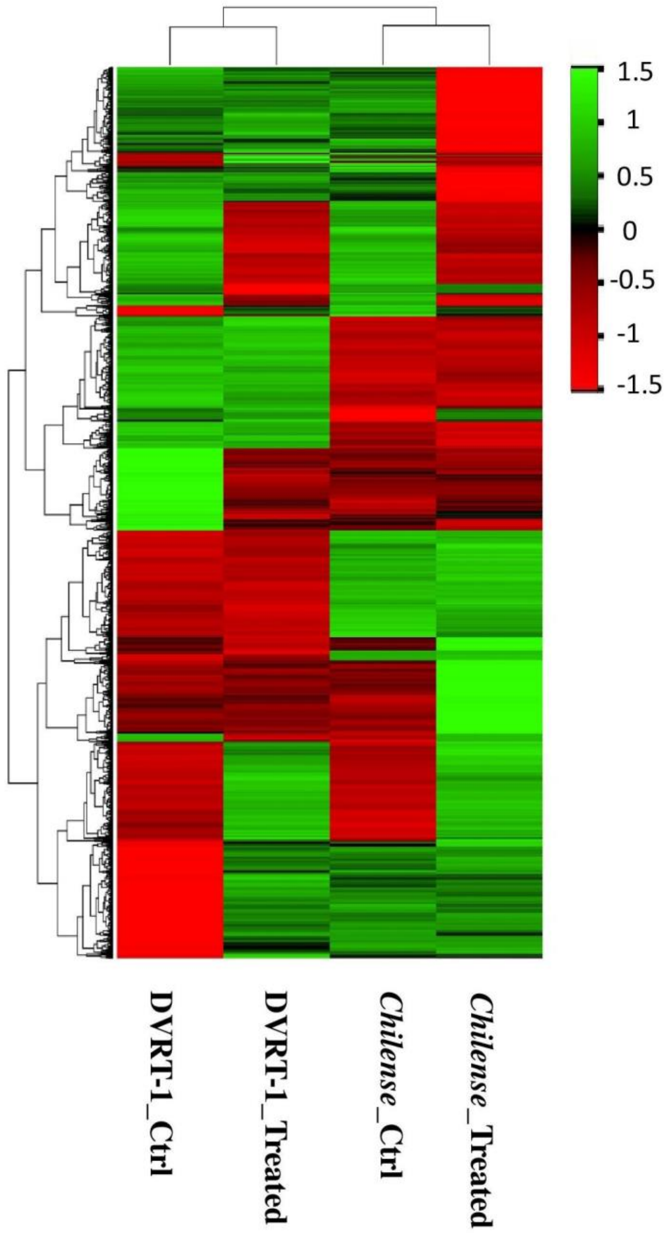

Supplemental Fig. S9

Gene ontology (GO) annotations of differentially expressed genes (DEGs) using Blast2GO program. DEGs were grouped into three main GO categories; biological process, cellular component and molecular function as well as into 27 subcategories.

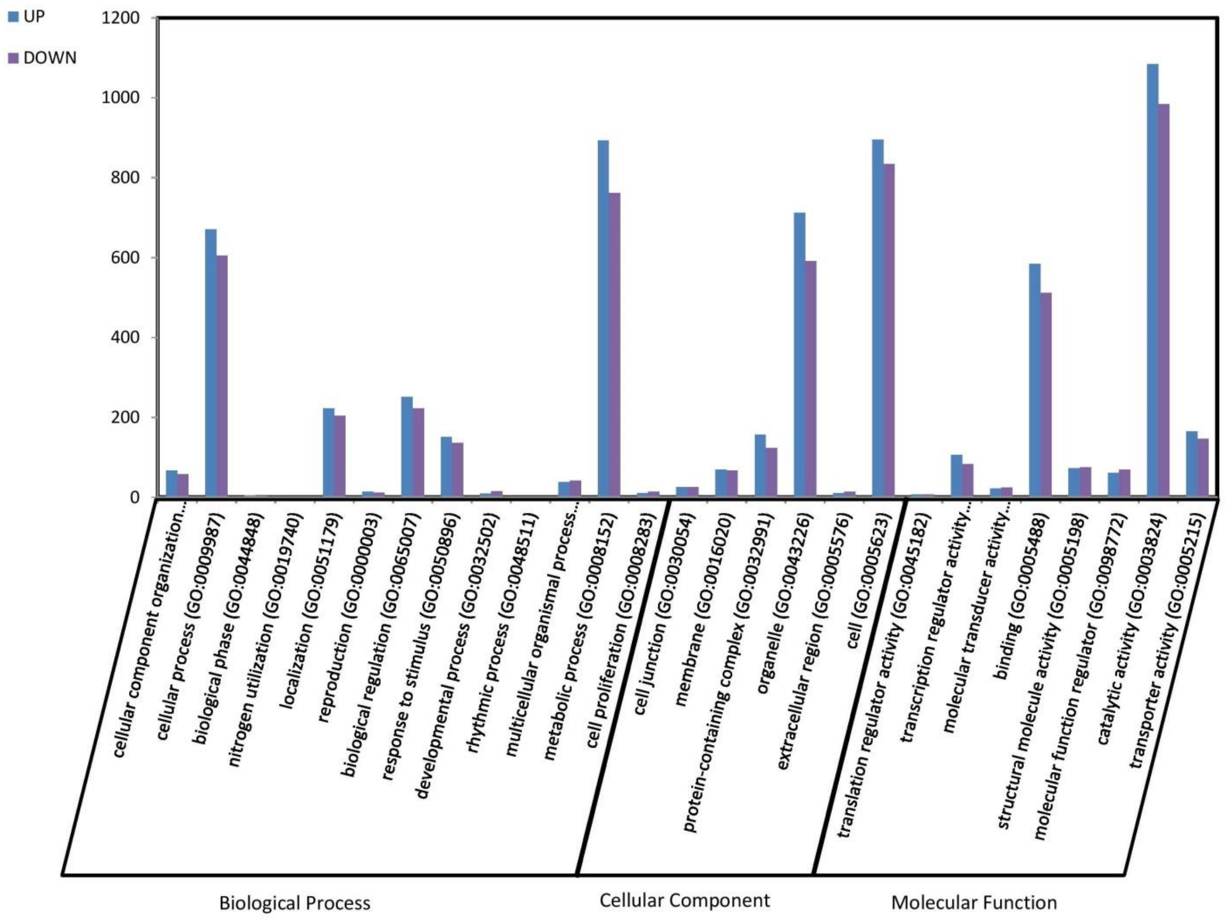

Supplemental Fig. S10

Pathway analysis of differentially expressed genes (DEGs). PANTHER14.1 tool was used for identification of pathway of DEGs. A total of 30 pathways were identified and among these pathways, the "Wnt signaling pathway" was over-represented and has not been reported previously with response to the salinity function.

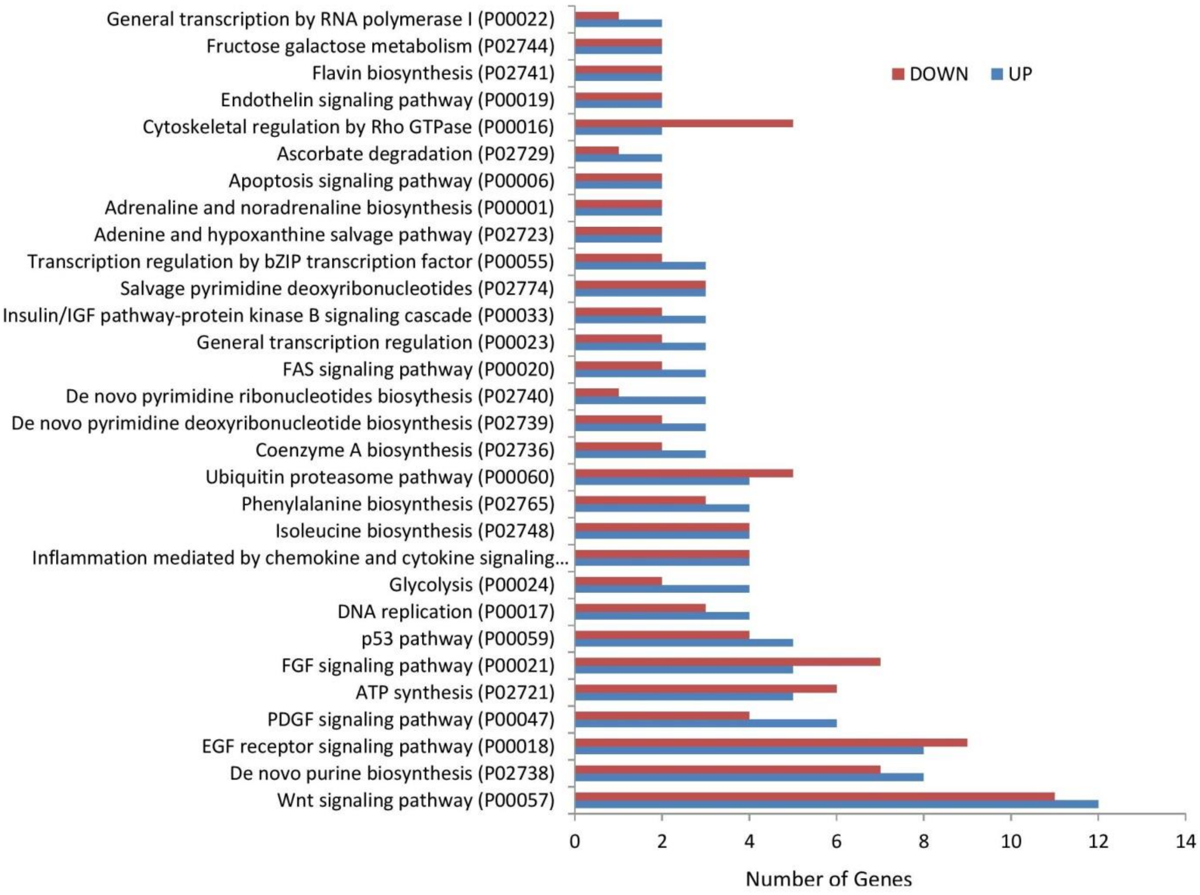

Supplemental Fig. S11a

Pathway analysis of assembled unigenes (*Chilense*_Control). A total of 115 pathways were identified, top 20 pathways were taken *Chilense*_Control sample for Pie chart representation.

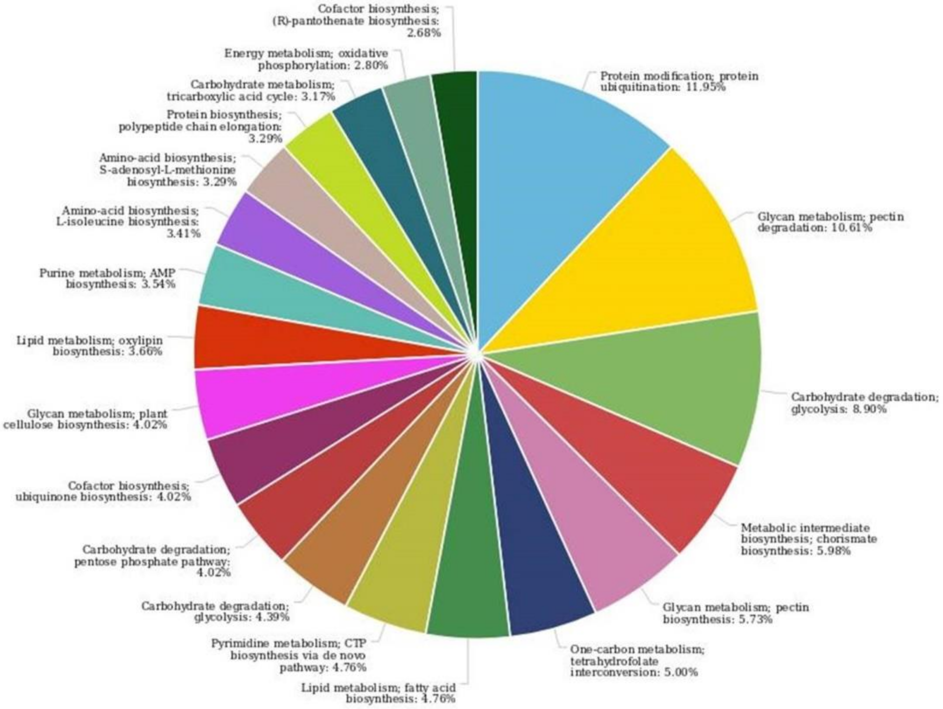

Supplemental Fig. S11b

Pathway analysis of assembled unigenes (*Chilense*_Treated). A total of 131 pathways were identified, top 20 pathways were taken *Chilense*_Treated sample for Pie chart representation.

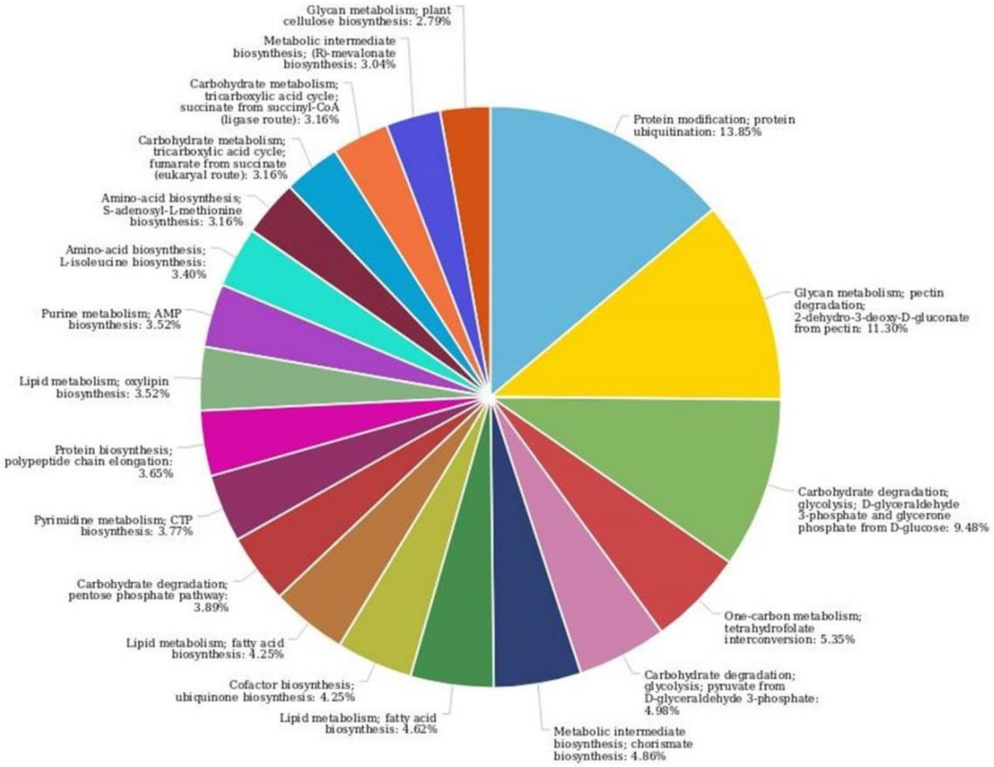

Supplemental Fig. S11c

Pathway analysis of assembled unigenes (DVRT-1 Control). A total of 137 pathways were identified, top 20 pathways were taken DVRT-1 Control sample for Pie chart representation.

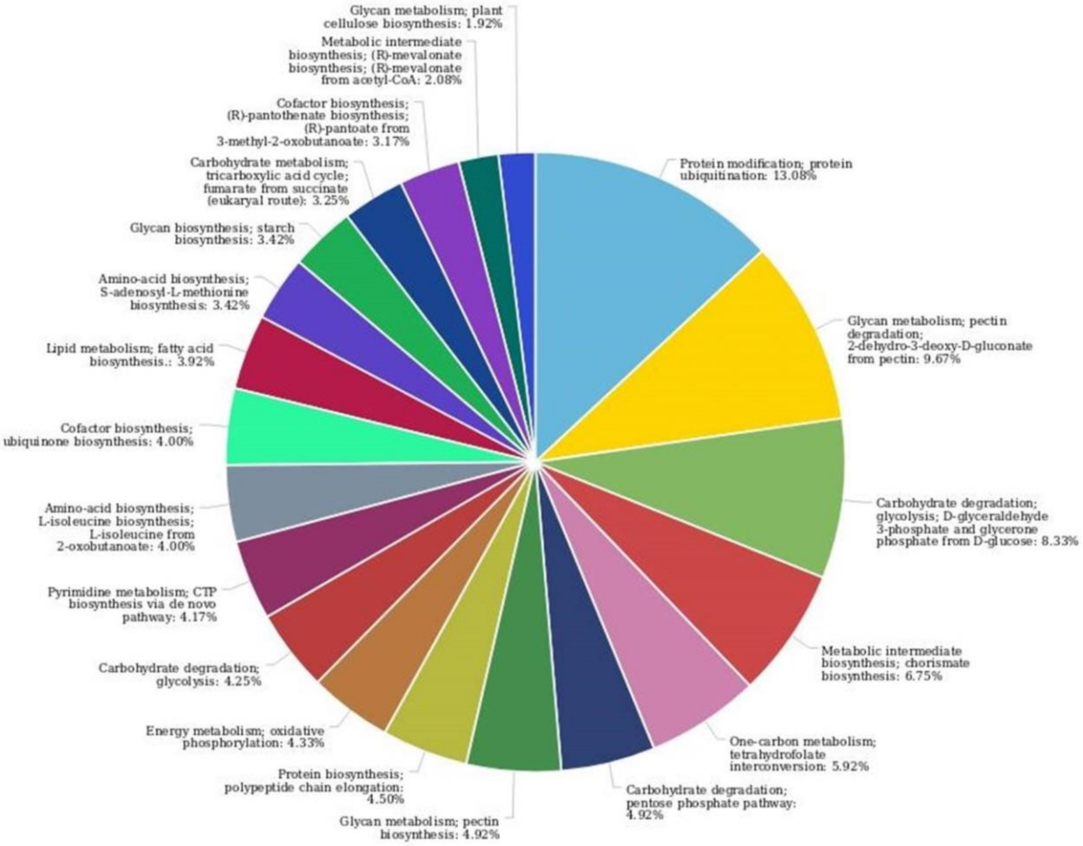

Supplemental Fig. S11d

Pathway analysis of assembled unigenes (DVRT-1 Treated). A total of 123 pathways were identified, top 20 pathways were taken DVRT-1 Treated sample for Pie chart representation.

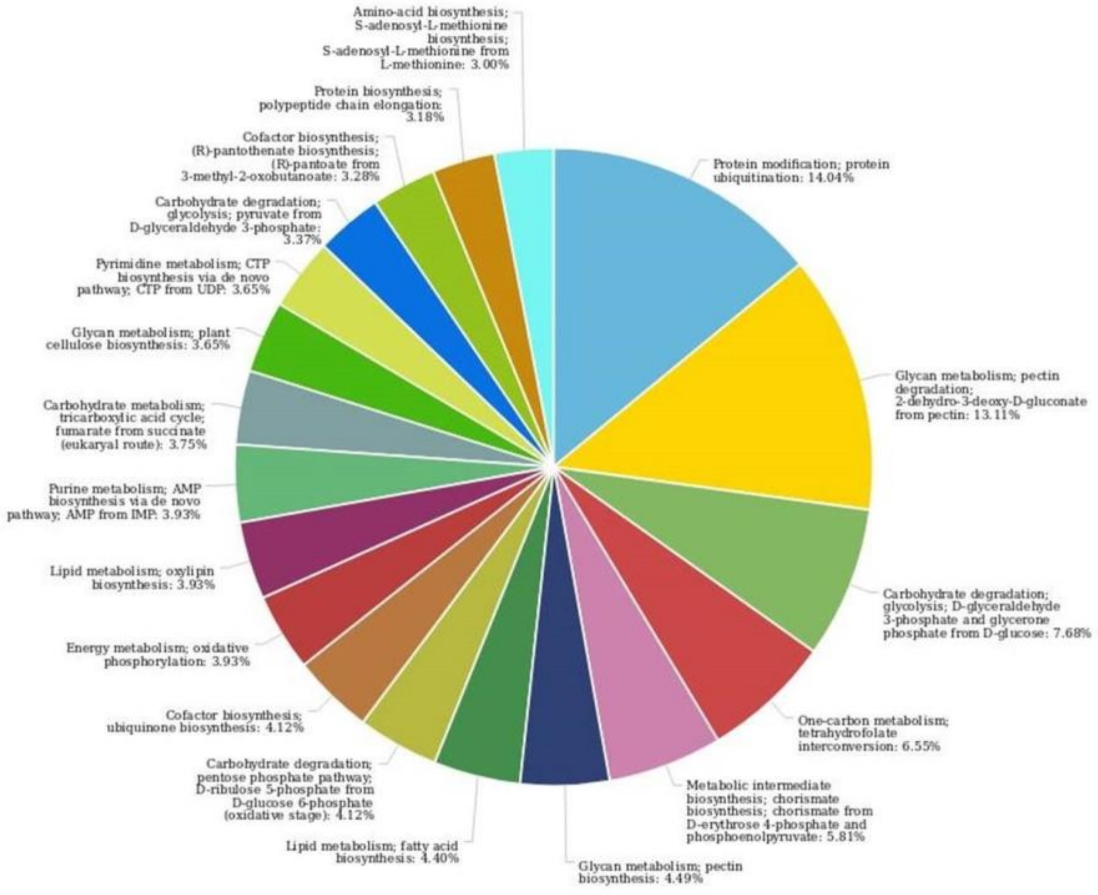

Supplemental Fig. S12

Expression patterns of selected candidate genes related with transporters, ROS scavenging and signaling transduction in salt treated and untreated *Chilense* and DVRT-1 determined by RNA-seq and qPCR. The RNA-seq values represent the ratio of the expression level in *chilense* to the expression level in DVRT-1. Bars with distinct letters are significantly different at P ≤ 0.05 applying the DMRT test.

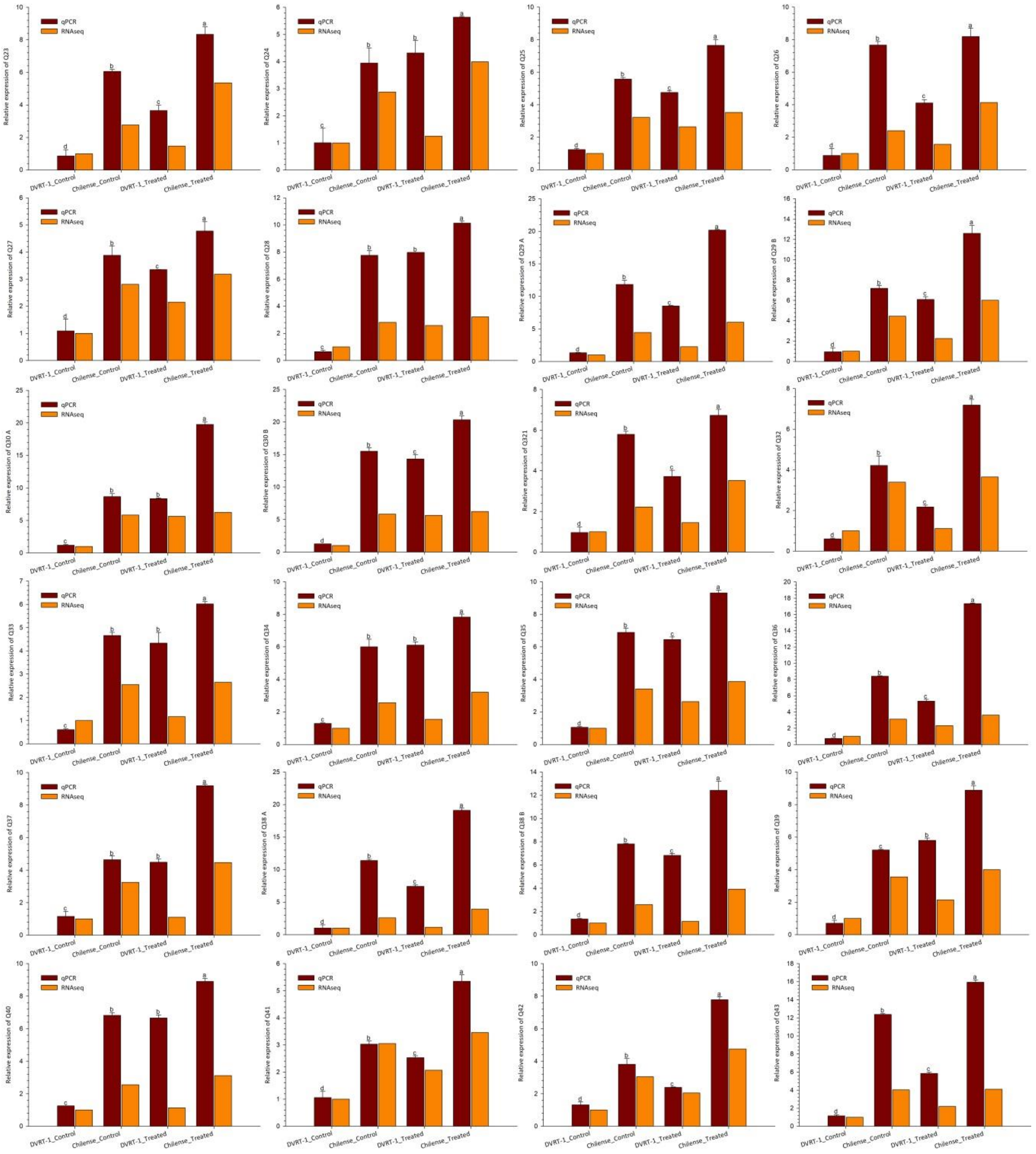

Supplemental Fig. S13

Expression patterns of selected candidate genes related with osmotic regulation, defence-stress, homeostasis, transporters and transcription factor in salt treated and untreated *Chilense* and DVRT-1 determined by RNA-seq and qPCR. The RNA-seq values represent the ratio of the expression level in *chilense* to the expression level in DVRT-1. Bars with distinct letters are significantly different at $P \leq 0.05$ applying the DMRT test.

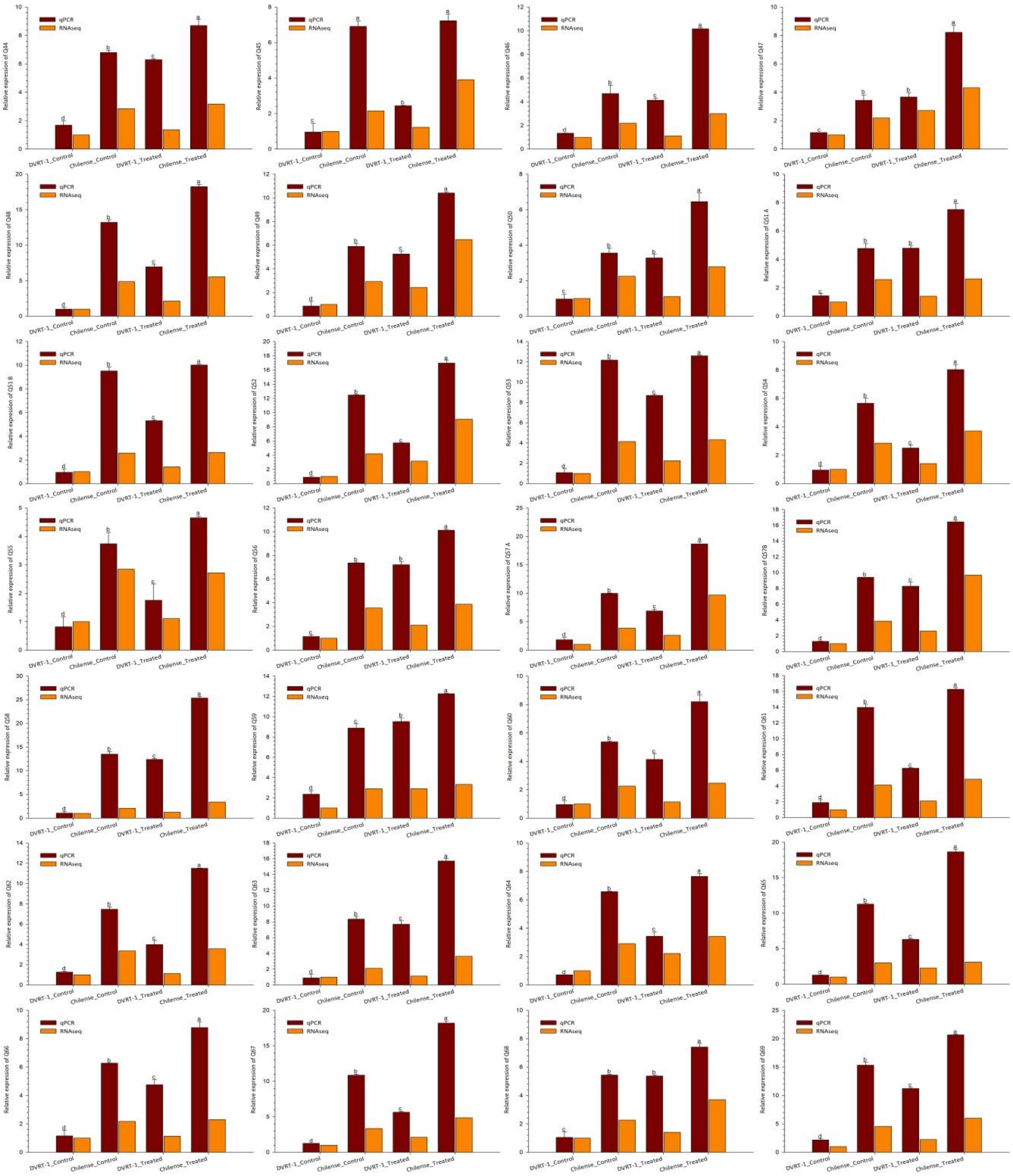

Supplemental Fig. S14

Picture depicting the salt sensitivity of the *S. lycopersicum* cv. DVRT-1 and salt tolerance of *S. chilense*; the salt stress was imposed by adding NaCl for 21 days. A non-stress or control treatment (0 days) was also carried out without NaCl with EC of 3.8 dSm^{-1} whereas; saline treatments received 500 mmol of NaCl for 21 days with EC of 26.8 dSm^{-1}, respectively.

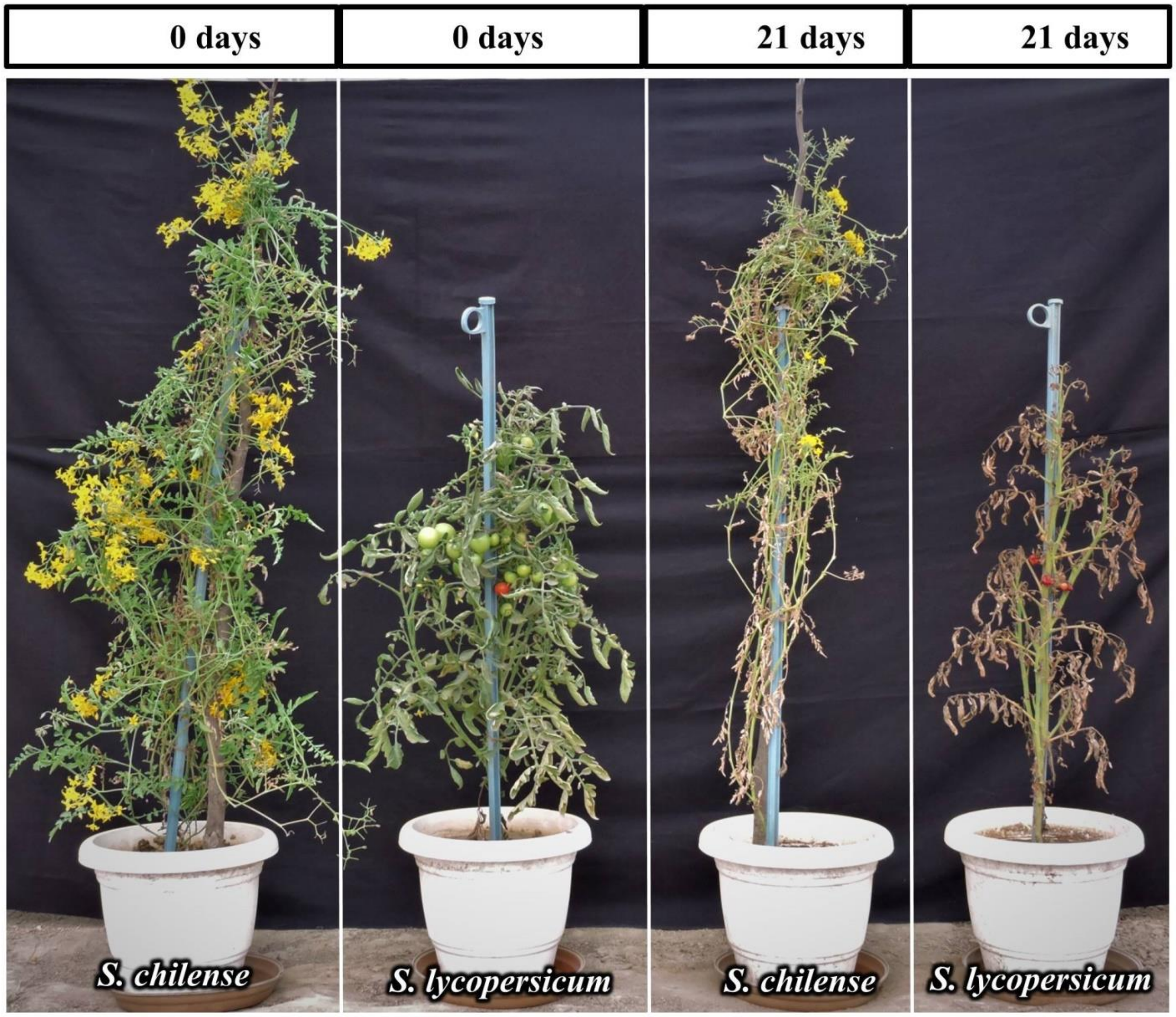

Supplemental Table S1

Summary of transcriptome sequencing data

Sample Name	Raw Reads	Clean reads	Clean bases	Clean bases (GB)	Error rate (%)	Q20 (%)	Q30 (%)	GC content (%)
*Chilense*_Control	88, 370, 504	87, 087, 908	12, 422, 293, 202	12.42	1.45	98.47	95.98	45.63
*Chilense*_Treated	101, 231, 584	100, 016, 490	14, 436, 367, 536	14.44	1.20	98.59	96.28	43.72
DVRT-1_Control	101, 509, 668	100, 085, 760	14, 190, 531, 518	14.19	1.40	98.53	96.17	46.43
DVRT-1_Treated	100, 283, 628	98, 820, 992	13, 918, 207, 430	13.92	1.46	98.66	96.43	44.13
Total	391, 395, 384	386, 011, 150	54, 967, 399, 686					

Supplemental Table S2

Summary of trancsriptome assembly quality

Transcriptome Assembly	*Chilense*_Control	*Chilense*_Treated	DVRT-1Control	DVRT-1Treated	Unigenes
Total length (nt)	107,039,157 (107.03 MB)	120, 019, 568 (120.01 MB)	143, 241, 095 (143.24 MB)	172, 656, 865 (172.7 MB)	304, 512, 139 (304.51 MB)
Total number	159, 618	177, 279	236, 319	236, 853	514, 747
N50	789	792	618	910	578
N90	336	337	331	345	325
Max length (nt)	21, 918	22, 865	46, 034	45, 439	46, 034
Min length (nt)	300	300	300	300	300
Mean length (nt)	412.591	413.124	369.567	472.901	591.576
300–500 bp	99, 461	110, 974	155, 345	137, 447	35, 4571
500–1000 bp	35, 695	39, 360	54, 988	58, 922	109, 215
1000–2000 bp	17, 593	18, 695	20, 199	27, 315	35, 629
> 2000 bp	7, 077	8, 496	6, 138	13, 507	16, 082

Supplemental Table S5

Distribution of simple sequence repeats (SSRs) from assembled unigenes. A total of 1,06,239 potential SSRs were identified, which distributed among 81,256 unigenes, and including 18,593 unigenes with more than one SSR.

Types	Number
Total number of sequences examined	514, 747
Total size of examined sequences (bp)	304, 512, 139
Total number of identified SSRs	106, 239
Number of SSR containing sequences	81, 256
Number of sequences containing more than 1 SSR	18, 593
Number of SSRs present in compound formation	8, 784
Number of mono-nucleotide SSR	69, 643
Number of di-nucleotide SSR	9, 062
Number of tri-nucleotide SSR	7, 123
Number of tetra-nucleotide SSR	629
Number of penta-nucleotide SSR	104
Number of hexa-nucleotide SSR	64

Supplementary Table Legends:

Supplemental Table S3: Kyoto encyclopedia of Genes and Genomes (KEGG) annotation of assembled unigenes. The unigenes numbers are listed with the Ko ID. KO_Descriptionand, KO_Group and KO_Family are shown in column C, D and E, respectively.

Supplemental Table S4: Karyotic Orthologous Groups (KOG) functional classification of assembled unigenes. A total of 1,26,937 unigenes were assigned into 26 diverse functional categories.

Supplemental Table S6: Details of simple sequence repeats (SSRs) from assembled unigenes. The unigenes with serial numbers are listed with the SSR repeat units. The repeat type and size are shown in column C, D and E. The number where the SSR repeat begins and ends are shown in the last two columns.

Supplemental Table S7: Transcription factors families analysis of assembled unigenes. Plant TFDB tool was used for identification of potential transcription factors families. A total of 57 transcription factor families, containing 6,353 unigenes were identified.

Supplemental Table S8: Differentially expressed genes (DEGs) summary report.

Supplemental Table S9: List of differentially expressed genes (DEGs) in *Chilense*_Treated vs DVRT-1_Treated.

Supplemental Table S10: List of differentially expressed genes (DEGs) in *Chilense*_Treated vs Control.

Supplemental Table S11: List of differentially expressed genes (DEGs) in DVRT-1_Treated vs Control.

Supplemental Table S12: Pathway analysis of assembled unigenes in *Chilense*_Control, *Chilense*_Treated, DVRT-1 Control and DVRT-1 Treated sample.

Supplemental Table S13: Gene Ontology (GO) enrichment analysis of up-regulated differentially expressed genes in different treatment and control groups using REVIGO based on the lowest p values.

Supplemental Table S14: Primer sequences used for qRT-PCR analysis and NormFinder stability check value of reference genes. The primer names with sequences are shown. The unigene sequences obtained by transcriptome analysis were used to design primers with the help of http://www.ncbi.nlm.nih.gov/tools/primerblast/ online tool.
